# Catalytic Oxidation of Methane to Oxygenated Products: Recent Advancements and Prospects for Electrocatalytic and Photocatalytic Conversion at Low Temperatures

**DOI:** 10.1002/advs.202001946

**Published:** 2020-10-27

**Authors:** Md. Selim Arif Sher Shah, Cheoulwoo Oh, Hyesung Park, Yun Jeong Hwang, Ming Ma, Jong Hyeok Park

**Affiliations:** ^1^ Department of Chemical and Biomolecular Engineering Yonsei University 50 Yonsei‐ro, Seodaemun‐gu Seoul 03722 Republic of Korea; ^2^ Department of Energy Engineering School of Energy and Chemical Engineering Low Dimensional Carbon Materials Center Perovtronics Research Center Ulsan National Institute of Science and Technology (UNIST) Ulsan 44919 Republic of Korea; ^3^ Clean Energy Research Center Korea Institute of Science and Technology (KIST) Seoul 02792 Republic of Korea; ^4^ Shenzhen Institutes of Advanced Technology Chinese Academy of Sciences Shenzhen Guangdong 518055 China

**Keywords:** electrocatalysis, methane oxidation, oxygenates, photocatalysis, product selectivity and low temperatures, stability

## Abstract

Methane is an important fossil fuel and widely available on the earth's crust. It is a greenhouse gas that has more severe warming effect than CO_2_. Unfortunately, the emission of methane into the atmosphere has long been ignored and considered as a trivial matter. Therefore, emphatic effort must be put into decreasing the concentration of methane in the atmosphere of the earth. At the same time, the conversion of less valuable methane into value‐added chemicals is of significant importance in the chemical and pharmaceutical industries. Although, the transformation of methane to valuable chemicals and fuels is considered the “holy grail,” the low intrinsic reactivity of its C—H bonds is still a major challenge. This review discusses the advancements in the electrocatalytic and photocatalytic oxidation of methane at low temperatures with products containing oxygen atom(s). Additionally, the future research direction is noted that may be adopted for methane oxidation via electrocatalysis and photocatalysis at low temperatures.

## Graphical abstract

Methane, a natural and potential greenhouse gas, is widely available. Conversion of methane can decrease global warming and warrant future energy and fuel supply. Herein, recent advancements of photocatalytic and electrocatalytic conversion of methane to oxygenates at low temperatures are summarized. Techniques to overcome challenges in methane oxidation are discussed. Prospects of methane oxidation by photocatalysis and electrocatalysis are demonstrated.

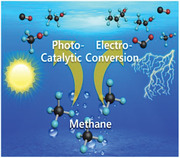

## 1 Introduction

**Figure 1:**
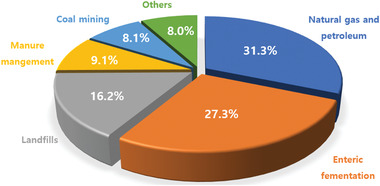
Distribution proportion of different CH_4_ sources.

Methane (CH_4_), as the simplest saturated hydrocarbon with the lowest C/H ratio,^[^ ^1^ ^]^ owns a high calorific value.^[^ ^2^, ^3^, ^4^ ^]^ The combustion of CH_4_ could release more energy per molecule of produced CO_2_ compared to oil (approximately CH_2_) or coal (approximately CH).^[^ ^5^, ^6^ ^]^ CH_4_ has been abundantly found on the earth, due to its high stability.^[^ ^7^ ^]^ Natural gas, one of the main existing form for CH_4_ (about 70–90% by volume), is plentiful in the crust around the world, constituting ≈21% of total principal energy sources on the earth. For instance, CH_4_ molecules could be held with water through hydrogen bonding in the form of methane hydrates (combustible ice), existing in the continental slopes of oceans, marine sediments, cold climate regions, and subsurface deposits.^[^ ^8^, ^9^ ^]^ The amount of methane hydrates has been estimated about (3000–20 000) × 10^12^ m^3^,^[^ ^10^, ^11^ ^]^ with energy calculated to be more than double of that from all other fossil fuels.^[^ ^12^ ^]^ Natural gas has been exploited from shale gas recently, the amount of which is estimated to be more than 7299 trillion cubic feet.^[^ ^13^, ^14^, ^15^, ^16^, ^17^ ^]^ Methane also could be naturally generated from biosystems as marsh gas through the anaerobic digestion of crops, wastes and residues.^[^ ^18^, ^19^ ^]^ Human activities, such as coal mining, natural gas or petroleum drilling and breakdown of garbage in landfills, significantly contribute to the release of CH_4_. The main existence form of CH_4_ could be classified in **Figure** **1**.

According to the abundant reserves, CH_4_ fuels possess superior economic efficiency with a high energy density (>1000 kWh m^−3^).^[^ ^20^ ^]^ CH_4_ could be burned directly with oxygen to obtain high amount of heat energy (Δ*H* ^0^_298K_ = −802 kJ mol^−1^),^[^ ^21^ ^]^ or catalytically oxidized to generate electrical energy in fuel cells.^[^ ^22^, ^23^ ^]^ Thus, as a cleaner energy compared to other conventional fuels,^[^ ^24^, ^25^, ^26^, ^27^, ^28^ ^]^ CH_4_ alone has the potential to compensate for the depletion of fossil fuel. However, it is a powerful greenhouse gas that exhibits a global radiative forcing of 0.61 W m^−2^, which amounts to ≈20% of all the greenhouse gases in 2011.^[^ ^29^, ^30^ ^]^ As the CH_4_ sources are mainly stored in depopulated areas, the exploitation and transportation of them to industrial areas are not economically viable.^[^ ^31^, ^32^ ^]^ In the atmosphere, the concentration of CH_4_ rose from 722 parts per billion (ppb) in the preindustrial era to 1867 ppb in 2018 with an over 2.5‐fold increase.^[^ ^6^ ^]^ The CH_4_ emission affects the earth's climate and global energy balance. Therefore, diminishing the leakage of CH_4_ is essential to decreasing global warming, pollution and climate change. Conversion of CH_4_ to value added chemicals is the most significant way to control its emission during the human activities. In this case, especially if converted at low temperatures, industrially important chemicals can be produced simultaneously. Unfortunately, most of the conversion processes are kinetically sluggish at low temperature due to the intrinsic inertness of CH_4_,^[^ ^21^, ^33^, ^34^ ^]^ requiring relatively high temperature (and pressure) to carry out these reactions.^[^ ^35^ ^]^

Up to now, numerous works have been done upon the conversion of CH_4_, with diversified review reports published. Most of the review works on CH_4_ conversion have involved several interests, such as partial oxidation over transition metal‐based catalysts,^[^ ^36^, ^37^, ^38^, ^39^ ^]^ oxidation of CH_4_ by heterogeneous catalysts,^[^ ^40^, ^41^ ^]^ metal organic framework‐based catalysts for CH_4_ oxidation,^[^ ^41^ ^]^ complete oxidation of CH_4_,^[^ ^7^, ^42^, ^43^, ^44^ ^]^ and electrocatalytic CH_4_ oxidation at high temperatures.^[^ ^36^, ^45^ ^]^ However, the theme of CH_4_ partial oxidation at low temperature is rare.^[^ ^46^ ^]^ Moreover, the fundamental chemical properties and the inertness origin for CH_4_ were neglected in those reviews. This review is devoted to the CH_4_ oxidation by electrocatalytic and photocatalytic processes at low temperatures, especially at room temperature.

In this review, we have discussed the chemistry behind the inertness of CH_4_, the significance for CH_4_ oxidization and challenges therein, and various processes for C—H activation with emphasis on electrocatalytic and photocatalytic routes, especially at room/low temperatures. The scope of this review is focused on the CH_4_ oxidation processes, involving with the cleavage of the C—H bonds and subsequent functionalization of the carbon atoms. For highlighting the particularity and professionality of this review, we aim at the well‐defined systems leading to the production of oxygenates from CH_4_ oxidation, excluding higher alkanes, halides or any other compounds without oxygen. At the end, we will outline the future research directions that researchers would like to adopt for efficient CH_4_ oxidation, which would be timely contribution to the energy conversion fields.

## 2 Intrinsic Motivation for CH_4_ Oxidation

### 2.1 The “Inert” Chemistry of CH_4_

**Figure 2:**
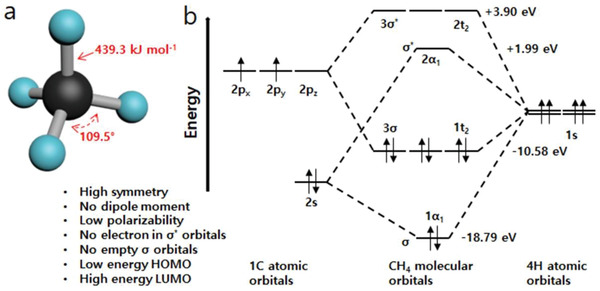
a) CH_4_ tetrahedral structure. b) Molecular orbital diagram with the corresponding energies, source: https://www.science.oregonstate.edu/~gablek/CH334/Chapter1/methane_MOs.htm.

At normal pressure and temperature, CH_4_ expresses colorless and odorless gas. It possesses tetrahedral molecular structure (**Figure** **2**a) consisting of four equivalent C—H bonds, according to the sp^3^ hybridization of the carbon atom (point group *T*_d_, C—H bond length of 1.087 Å and H—C—H bond angle of 109.5°).^[^ ^40^ ^]^ Due to the directional orientation, sp^3^ bonding orbitals are less well‐adapted to the formation of new bonds in the transition state, resulting in a barrier restricting the chemical reactions. The CH_4_ molecule has immense stability and symmetry with a small polarizability (2.84 × 10^−40^ C^2^ m^2^ J^−1^),^[^ ^47^ ^]^ due to the small electronegativity difference between carbon (2.55) and hydrogen (2.2).^[^ ^48^ ^]^ As a result, a comparatively high local electric field is needed to induce polarization and to allow electrophilic or nucleophilic attack of CH_4_ for initiating chemical reactions. Notably, the carbon atom in CH_4_ is slightly negatively charged (*δ*_C_ = −0.185), while the hydrogen atoms get a slightly positive charge (*δ*_H_ = +0.046).^[^ ^49^ ^]^ The CH_4_ molecule contains four bonding molecular orbitals (Figure 2b) that are formed by the overlap of the four valence orbitals of the central carbon atom and one valence orbital from each of the four hydrogen atoms.

CH_4_ has triply degenerate molecular orbitals produced as a result of the overlap of two 2p carbon orbitals and three 1s orbitals of the hydrogen atoms. In the ground electronic state (*X* ^1^ *A*_1_), all electrons occupy bonding molecular orbitals with an electronic configuration of (1*a*_1_)^2^(1*t*_2_)^6^(2*a*_1_)^0^.^[^ ^50^ ^]^ The absence of electrons in the antibonding molecular orbitals (σ*) guarantees the sturdy stability of CH_4_ molecule.^[^ ^51^ ^]^ Moreover, the absence of low‐lying empty orbitals (*σ* molecular orbitals) and high‐energy filled orbitals (σ* molecular orbitals) (Figure 2b) make the participation of CH_4_ in any chemical reaction tough.^[^ ^4^, ^52^ ^]^ The highest occupied molecular orbital (HOMO) in CH_4_ has low energy and the lowest unoccupied molecular orbital (LUMO) has high energy. As a result, it is challenging to remove electrons from the HOMO or to add them to the LUMO of CH_4_.^[^ ^47^, ^53^ ^]^ The absence of lone electron pairs that can be relatively easily attacked is another reason for the inertness of methane. Furthermore, CH_4_ is highly resilient to nucleophilic attack because electron donation to the high‐energy σ* molecular orbital is energetically unfavorable and sterically hindered.

Compared to the other alkane, CH_4_ contains four unusually strong localized C—H bonds (the bond energy of H—CH_3_ was calculated to be 439.3 kJ mol^−1^ under standard conditions).^[^ ^33^, ^54^, ^55^ ^]^ Therefore, C—H bond scissions for CH_4_ (both homolytic and heterolytic) are not feasible. In a contrast, methyl cations (CH_3_^+^) are the least stable carbocations, which make methane the least reactive alkane for the abstraction of hydride ions. Highly negative electron affinity (≈−1.9 eV) indicates CH_4_^−^ anion is less stable than CH_4_ itself.^[^ ^56^ ^]^ Additionally, CH_4_ possesses critically unreactive C—H bonds for any electron transfer reaction due to its high ionization energy (≈12.6 eV).^[^ ^57^ ^]^ The low proton affinity (543.9 kJ mol^−1^)^[^ ^58^ ^]^ and weak acidity (p*K*_a_ ≈ 48)^[^ ^59^ ^]^ restrict its activation by any acid or base. Nevertheless, removal of the electrons from the *σ* bonds in CH_4_ by strong electrophiles, although challenging, is somewhat facile. Homolytic cleavage of the C—H bonds, followed by the formation of hydrogen radical and methyl radical, is the most facile way for CH_4_ oxidation.^[^ ^60^ ^]^ However, CH_4_ should be a choice for reactions involving sufficiently hindered reagents due to its small size.

### 2.2 Challenges and Significances of CH_4_ Oxidation

Conversion of CH_4_ to its derivatives at low temperatures through direct pathways is kinetically challenging.^[^ ^61^ ^]^ For cleaving the C—H bonds and thereby oxidizing the molecule to any oxygenated products (except CO_2_), a large amount of energy usually should be provided. Often, this amount of energy is supplied from thermal energy by increasing the reaction temperature. In reality, to achieve CH_4_ oxidation, the reaction would always be operated at fairly high temperatures (>≈700 °C), which generally leads to reactions driven by a free radical mechanism with intrinsic low selectivity.^[^ ^40^ ^]^ High temperature conditions, always along with high pressures, indicate high costs for industrial applications, in addition to safety issues. The low solubility of gaseous CH_4_ (compared to the good solubility of the related oxidation products) imparts another challenge for its oxidation.^[^ ^46^ ^]^ Thus, the concentration of the oxidation products would be much higher than that of CH_4_ in any reaction mixture, accompanying with severe side reactions, which could generate serious selectivity issues for the products formation.

**Figure 3:**
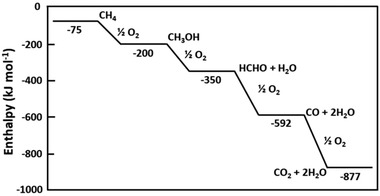
Changes in enthalpy at 298 K for successive oxidation of CH_4_. Reproduced with permission.^[^ ^62^ ^]^ Copyright 1991, Elsevier.

Other issues, such as the stability and durability of catalysts and the formation of CO_2_, should also be considered for CH_4_ oxidation at high temperatures and pressures. For highly efficient CH_4_ oxidation, noble metals (such as Pt, Ir, Ru, Rh, and Pd) are frequently employed as catalysts, which are expensive and rare. Thus, the valuable CH_4_ oxidation suffers highly energy demanding, expensive processes that are challenging for industries. In the meantime, the superior activity of CH_4_ oxidation products and other intermediates compared to CH_4_ itself (e.g., C—H bond dissociation energy in CH_3_OH is 0.4 eV less than that in methane) would cause excessive oxidation of CH_4_.^[^ ^62^, ^63^ ^]^ Naturally, stopping further oxidation is quite difficult even methanol has been formed locally by a catalyst at low temperatures,^[^ ^64^ ^]^ as depicted in **Figure** **3**. Therefore, kinetic protection and selective separation of the products are necessary to obtain the desired products, making the oxidation process more complex. These challenges in CH_4_ oxidation result in a small number of industrial applications.

**Figure 4:**
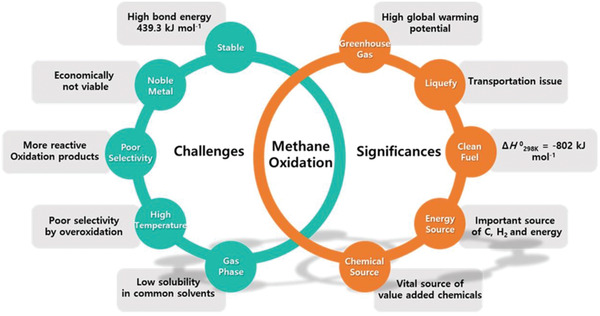
Challenges and significances of CH_4_ oxidation.

On the other hand, CH_4_ oxidation is important due to several reasons such as decreasing the global warming. CH_4_ can also be used as raw material to produce useful chemicals,^[^ ^65^, ^66^, ^67^, ^68^ ^]^ especially as hydrogen (energy density of 270 kWh m^−3^),^[^ ^20^ ^]^ which is considered as the cleanest and greenest energy source in addition to being one of the world's most important chemicals.^[^ ^69^, ^70^, ^71^ ^]^ Notably, ≈50% of the world's demand for hydrogen (almost 55 × 10^6^ ton per year) derives from natural gas.^[^ ^72^, ^73^ ^]^ CH_4_ is a vital source of valuable chemicals, such as methanol and CO. Moreover, CH_4_ could not be easily liquefied (critical temperature −82.3 °C) at practically accessible low temperatures,^[^ ^6^ ^]^ causing transportation problems in remote areas. The main challenges and significance of CH_4_ oxidation has been summarized in **Figure** **4**.

## 3 Processes for CH_4_ Oxidation

Partial oxidation of CH_4_ leads to the formation of value‐added chemicals that can be used in the chemical and energy sectors and the pharmaceutical industry. Therefore, much effort has been directed to partially oxidize CH_4_. For low temperature conditions, the conversion processes can be classified into electrochemical process,^[^ ^74^ ^]^ photocatalysis,^[^ ^75^, ^76^ ^]^ enzyme catalysis,^[^ ^77^, ^78^, ^79^ ^]^ chemical catalysis or reagent process,^[^ ^80^, ^81^ ^]^ and liquid phase oxidation (employing hydrogen peroxide).^[^ ^55^ ^]^ After comprehensive comparison, electrochemical and photocatalytic processes have performed promising prospect for industrial application at low temperatures, which will be described in details in the following sections.

### 3.1 Electrochemical Processes

**Figure 5:**
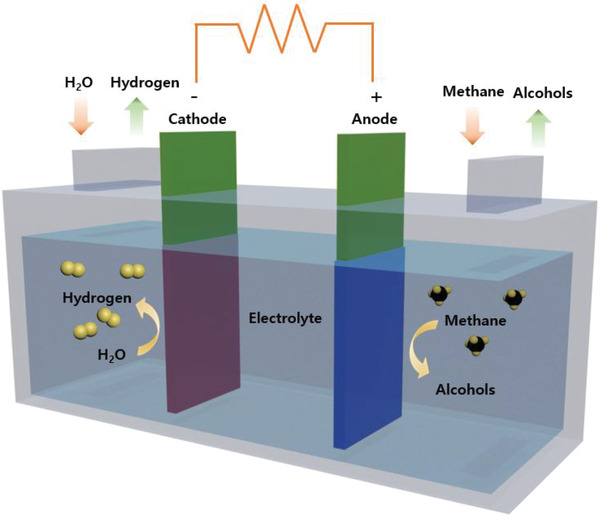
Diagram of an electrochemical cell for CH_4_ oxidation to alcohols.

Electrochemistry is a powerful technique to control redox reactions through the precise bias application, offering electrochemical oxidation as an economical alternative route to chemical conversion processes.^[^ ^82^, ^83^, ^84^ ^]^ The advantages of the electrochemical processes could be summarized as, i) relatively low cost (neglecting the initial cost of the equipment, power is generally less expensive than chemical reagents) with adequate yield,^[^ ^85^ ^]^ ii) usually occurring under ambient conditions with low temperatures (≤100 °C),^[^ ^86^, ^87^ ^]^ iii) convenient for scaling up,^[^ ^21^, ^88^ ^]^ and iv) satisfaction of most principles for green chemistry.^[^ ^89^, ^90^ ^]^ More importantly, in electrochemical process, kinetics and therefore the rate of product generation and their selectivity can be controlled by monitoring the applied potential.^[^ ^91^ ^]^ In contrary, thermal conversion of methane (reforming of methane) occurs at high temperatures (>≈700 °C) that impart several challenges as discussed in Section 2.2. Moreover, at high temperatures catalyst degrades rapidly due to coking^[^ ^92^ ^]^ and catalyst sintering, and greenhouse gas emission occurs.^[^ ^93^ ^]^ On the other hand, enzymatic oxidation (e.g., by methane monooxygenases) of methane to methanol occurs under ambient conditions in the presence of O_2_.^[^ ^94^ ^]^ However, such oxidation suffers from poor kinetics and low carbon and energy efficiencies.^[^ ^95^ ^]^ Moreover, these enzymes themselves are usually slow and complex. Over the last decades, electrochemical oxidation of CH_4_ has been a topic of principal interest.^[^ ^96^ ^]^ Electrochemical CH_4_ oxidation is thermodynamically favorable at modest potentials (e.g., CH_4_ (g) + H_2_O (l) → CH_3_OH (aq) + 2H^+^ + 2e^−^ reaction has *E* ^0^ = 0.586 V versus normal hydrogen electrode (NHE) at pH = 0 and 298 K), but kinetically sluggish, which could be improved with the high overpotential assistance for proceeding at low temperatures (**Table** **1**).^[^ ^97^ ^]^ However, at high overpotentials (especially at ≥0.8 V vs reversible hydrogen electrode (RHE)), water oxidation (oxygen evolution) undergoes at high rate,^[^ ^98^, ^99^ ^]^ resulting in extra energy loss as well as increased cost of CH_4_ oxidation significantly. In the electrochemical processes, either oxygen site on the electrode surface or free radicals at the electrode/electrolyte interface can activate CH_4_ at ambient temperatures.^[^ ^91^ ^]^ Schematic diagram of an electrochemical cell for CH_4_ oxidation is presented in **Figure** **5**. It consists of three main parts: i) an anode, where CH_4_ oxidation occurs; ii) a cathode, where the reduction half‐reaction takes place; and iii) an electrolyte, an ion conducting medium that separates the two electrodes. The anode and cathode are connected through an external circuit. However, in this process selective production of a desired product is challenging as the electrode potential values of different products are often very close and high reactivity of free radicals and reactive oxygen species.^[^ ^100^ ^]^

**Table 1:** Methane oxidation to oxygenates and the respective potentials. The data were adapted with permission;^[^ ^97^ ^]^ (aq) indicates aqueous solution

Methane oxidation reaction	*E* (V vs RHE)
CH_4_(g) + H_2_O(l) → CH_3_OH(aq) + 2H^+^(aq) + 2e^−^	0.58
CH_4_(g) + H_2_O(l) → HCHO(aq) + 4H^+^(aq) + 4e^−^	0.46
CH_4_(g) + H_2_O(l) → HCOOH(aq) + 6H^+^(aq) + 6e^−^	0.26
CH_4_(g) + H_2_O(l) → CO(g) + 6H^+^(aq) + 6e^−^	0.26
CH_4_(g) + H_2_O(l) → CO_2_(g) + 8H^+^(aq) + 8e^−^	0.17

In the following few paragraphs we will discuss fundamental parameters that play important role in CH_4_ electrooxidation reactions.

Onset potential: It is an intrinsic property of any electrocatalytic reaction. Ideally, onset potential of a reaction should be equal to its equilibrium potential. However, in practice onset potential is usually much greater than the equilibrium potential, as any electrode reaction occurs only after surmounting the electrode kinetic energy barrier. It is often difficult to find out the exact value of onset potential. Therefore, often it is measured at a particular current density, e.g., 1 mA cm^−2^.

Current density: It is an important parameter to measure the efficiency of a catalyst. It is usually expressed as mA cm^−2^. However, current density could be measured by the unit mass of the catalyst on the electrode surface.

Faradaic efficiency (FE): Faradaic efficiency or current efficiency is the efficiency with which electrons carry out a desired electrochemical reaction. It can be calculated by the following equation. FE(%) = *αnF*/*Q*, where, *α* is the number of electrons transferred in a desired electrochemical reaction and *n* is the number of mole(s) of the product formed (e.g., for MeOH production from CH_4_, *α* is 2 and *n* is 1, see Table 1), *F* is the Faraday constant, 96 485 C mol^−1^, and *Q* is the number of charge passed. Faradaic efficiency is a measure of product selectivity.

#### 3.1.1 Electrochemical Systems for CH_4_ Oxidation

Most conversion processes at low temperatures are ideal and feasible because the energy input is low and reaction conditions can be easily achieved and maintained. **Table** **2** summarizes literatures for electrochemical CH_4_ oxidation at low temperatures. In early studies, electrocatalytic CH_4_ oxidation were carried out on platinum electrodes at an appreciable rate in aqueous phosphoric, sulfuric and perchloric acid at 60–150 °C.^[^ ^101^, ^102^, ^103^, ^104^ ^]^ However, noble metal Pt restricts its wide applications. Fortunately, Frese replaced Pt by gold, glassy carbon, copper and mercury cathodes for partial oxidation of CH_4_ in different aqueous electrolytes (e.g., 0.01–2 m KOH, 2 m NaOH, and 0.1 m NaClO_4_ solution) containing dissolved oxygen in the potential range of 0.8–0.4 V (vs dynamic hydrogen electrode) at 25 °C.^[^ ^105^ ^]^ The main oxidation product was formaldehyde, although a little amount of methanol was produced in some experiments. These products were generated at high rates due to the reactive oxygen species (e.g., O_2_^−^ or O_2_H^−^) that were formed as a stable intermediate by electrochemical reduction of oxygen.

**Table 2:** Summaries of electrocatalytic CH_4_ oxidation systems at low temperatures

Catalyst	*T* [°C]	Oxidant	Electrolyte [m]	Potential [V]	Products	Mechanism
Au, glassy carbon, Hg, Cu	25	O_2_	KOH (0.01–2), NaOH (2), NaClO_4_ (0.1)	0.8–0.4 (DHE)^a)^	HCHO, MeOH, CO and CO_2_	Radical ^[^ ^105^ ^]^
CNT/Nafion/Ni(OH)_2_/Ni^b)^	RT	NaOH	NaOH (1.0)	0.27 (Ag/AgCl)	MeOH	^[^ ^106^ ^]^
TiO_2_/RuO_2_/PTFE/V_2_O_5_ ^c)^	RT	RuO_2_/V_2_O_5_	Na_2_SO_4_ (0.2)	2.0 (SCE)	MeOH, HCHO, HCOOH	Radical ^[^ ^108^ ^]^
NiO–ZrO_2_	RT	CO_3_^2−^	Na_2_CO_3_ (0.1)	0.5–0.6 (SCE)	MeOH, EtOH, CO, Pr*^i^*OH, CH_3_COCH_3_	^[^ ^74^ ^]^
Pt	RT	Cl_2_	KCl (0.6)	1.0–1.3 (SCE), Hg lamp	MeOH, CH_3_Cl, CH_2_Cl_2_, CHCl_3_	Radical ^[^ ^109^ ^]^
Pt	RT	Cl^−^	KCl (pH 11)	1.3 (SCE)	MeOH, CH_3_Cl	Radical ^[^ ^110^ ^]^
Co_3_O_4_/ZrO_2_	RT	CO_3_^2−^	Na_2_CO_3_ (0.5)	2.0	MeOH, HCHO, EtOH, PrOH, CH_3_CHO	Radical ^[^ ^111^ ^]^
Pt/C, Pt/C‐ATO, Pd/C, Pd/C‐ATO^d)^	25, 80	ATO	H_2_SO_4_ (0.5)	0.9 (RHE)	CO_2_	^[^ ^112^ ^]^
Pd‐black/VO(acac)_2_‐VGCF cathode^e)^	25	O_2_	H_3_PO_4_ (1)	0.65 V	CO_2_	Radical ^[^ ^113^, ^114^ ^]^
V_2_O_5_/SnO_2_ anode	100	H_2_O	Sn_0.9_In_0.1_P_2_O_7_	900 mV	MeOH	Radical ^[^ ^115^ ^]^
ZrO_2_:NiCo_2_O_4_	25	CO_3_^2−^	0.5 m Na_2_CO_3_	2.0	PrOOH, AcOH,^f)^ acetone	^[^ ^116^ ^]^
NiO/Ni	25	NaOH	0.1 m NaOH	1.40 (RHE)	EtOH	^[^ ^117^ ^]^
NiO@Ni hollow fiber	25	NaOH	0.1 m NaOH	1.46 (RHE)	MeOH and EtOH	^[^ ^118^ ^]^

^a)^ Dynamic hydrogen electrode;

^b)^ Multiwalled carbon nanotube;

^c)^ Polytetrafluoroethylene;

^d)^ Antimony‐doped tin oxide;

^e)^ Vertically grown carbon nanofiber;

^f)^ Acetic acid.

For avoiding the toxic materials, Qiao et al. demonstrated electrocatalytic CH_4_ oxidation over multiwalled carbon nanotube‐Nafion/nickel hydroxide‐modified nickel electrodes in 1.0 m NaOH solution.^[^ ^106^ ^]^ A linear relationship could be observed between the oxidation peak current and the concentration of CH_4_, which indicated that CH_4_ was effectively oxidized to methanol by NiOOH electrocatalyst. In an interesting work focusing on the electrode design, Rocha et al. developed a gas diffusion electrode (GDE) for the selective methanol electrosynthesis from CH_4_ on a V_2_O_5_ incorporated TiO_2_/RuO_2_/polytetrafluoroethylene (PTFE) electrode.^[^ ^107^ ^]^ Formaldehyde and formic acid were synthesized at the similar rate making the process poor selective for methanol formation. Nevertheless, increasing potential increased the amount of methanol, which reached at a maximum of 30% current efficiency at 2.2 V versus saturated calomel electrode (SCE). In a follow up work, the same group reported that incorporation of V_2_O_5_ into TiO_2_/RuO_2_/PTFE GDE suppressed the formation of formaldehyde and formic acid, thereby increased MeOH selectivity.^[^ ^108^ ^]^ A 5.6 wt% V_2_O_5_ loading to the GDE increased the current efficiency of the electrode to 57% at 2.0 V versus SCE. In the catalytic process, CH_4_ was first transformed to methyl bisulfate (by the supporting electrolyte, Na_2_SO_4_), which was then hydrolyzed to methanol by the vanadium redox couples. The electrocatalytic experiment was carried out for 1 h. However, the catalyst stability was not reported.

#### 3.1.2 Electrolyte Mediated CH_4_ Oxidation

**Figure 6:**
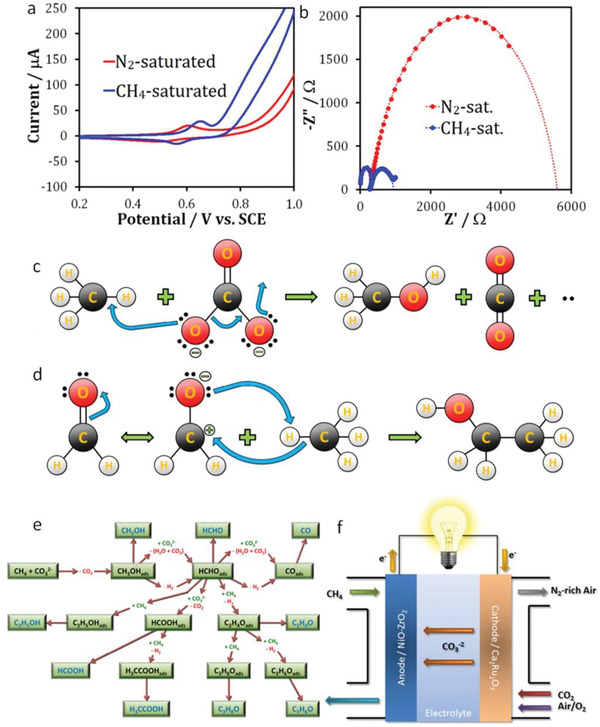
a) Cyclic voltammograms and b) *iR*‐corrected Nyquist plots for N_2_‐ and CH_4_‐saturated 0.1 m Na_2_CO_3_ solution over a NiO–ZrO_2_ electrocatalyst. c) Reaction mechanism for the formation of methanol from CH_4_ and carbonate ions. d) Formation of the C—C bond in ethanol. e) Proposed reaction pathways for the activation of CH_4_ by carbonate ions. f) The electrochemical cell that was constructed in this work. Reproduced with permission.^[^ ^74^ ^]^ Copyright 2013, The Electrochemical Society.

Hydroxide ions are conventionally used in the electrochemical CH_4_ oxidation as alkaline medium, which not only serve the purpose of a base but also act as an oxidizing agent. Unfortunately, OH^−^ ions do not have enough oxidizing ability to abstract protons from the unusually strong C—H bonds in CH_4_, especially at low temperatures. This is the reason for which CH_4_ oxidation in hydroxide media does not show appreciable activity.^[^ ^119^ ^]^ Therefore, Spinner and Mustain reported electrochemical CH_4_ activation in Na_2_CO_3_ (0.1 m) aqueous solution at room temperature on a NiO–ZrO_2_ composite catalyst.^[^ ^74^, ^120^ ^]^ The carbonate ions were adsorbed on the nonconducting ZrO_2_, while CH_4_ was adsorbed and activated by NiO. Oxygen ions were then abstracted from the carbonate ions and donated to the electrochemically active sites to make new bonds with carbon or hydrogen in CH_4_. It is interesting to note that, unlike OH^−^ ions, CO_3_^2−^ ions donate oxygen ions with successive release of CO_2_, which generated a large change in the enthalpy of reaction, favoring oxidation kinetics even at low temperatures.^[^ ^111^ ^]^ Additionally, the presence of the redox couple Ni^2+^/Ni^3+^ (by the reaction, Ni(OH)_2_ + OH^−^ ↔ NiOOH + H_2_O + e^−^) at 0.5–0.6 V vs SCE favored the oxidation process. The products were identified as different oxygenates, such as methanol, ethanol, isopropanol, formaldehyde, formate, acetate, acetone, and carbon monoxide. Oxygen and carbon dioxide were also formed slightly from the electrolysis of carbonate ions and/or the oxygen evolution reaction, which was catalyzed by NiOOH above 0.85 V vs SCE (by the reaction 4OH^−^ → O_2_ + 2H_2_O + 4e^−^). **Figure** **6** shows the CV and *iR*‐corrected Nyquist plots for the electrooxidation on the NiO–ZrO_2_ composite catalyst in N_2_‐ and CH_4_‐saturated CO_3_^2−^ solutions. The increased current density and decreased resistance (≈87%) in the CH_4_‐saturated electrolyte compared to the N_2_‐saturated one demonstrated the CH_4_ oxidation (Figure 6a,b). The authors assumed that the Ni^3+^ species (NiOOH) was responsible for the oxidation of the intermediate oxygenates.^[^ ^120^, ^121^, ^122^ ^]^ Figure 6c,d shows the proposed mechanism for the formation of methanol and the C—C bonds in ethanol, respectively. Interestingly, in this work new C—C bonds were formed. The suggested reaction pathways for the formation of different products are depicted in Figure 6e. The electrochemical device constructed is displayed in Figure 6f. In a closely related work, Mustain and co‐workers, demonstrated the essential role of ZrO_2_ for CH_4_ conversion in a low temperature electrochemical process with the carbonate cells and proved that carbonate ions donated oxygen ions.^[^ ^123^ ^]^ In contrast, with hydroxide‐based cells, the catalyst nickel oxyhydroxide itself functions as an oxygen donor for the CH_4_ conversion reaction.

**Figure 7:**
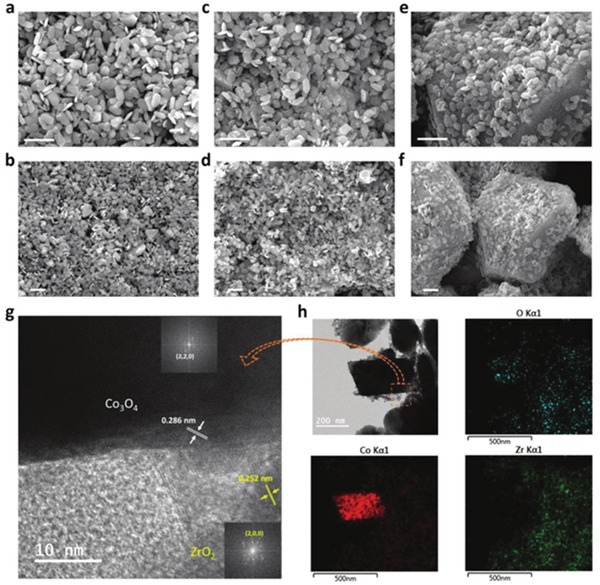
SEM micrographs of the a,b) 1–2 ZrO_2_/Co_3_O_4_, c,d) 1–4 ZrO_2_/Co_3_O_4_, and e,f) 1–6 ZrO_2_/Co_3_O_4_ electrocatalysts. The scale bars in (a–f) are 1 µm. g) High‐resolution (HR)‐TEM image. The insets in (g) are fast Fourier transform patterns. h) TEM micrograph and elemental mappings of O, Co, and Zr in the 1–4 ZrO_2_/Co_3_O_4_ electrocatalyst. Reproduced with permission.^[^ ^111^ ^]^ Copyright 2017, Wiley‐VCH GmbH.

**Figure 8:**
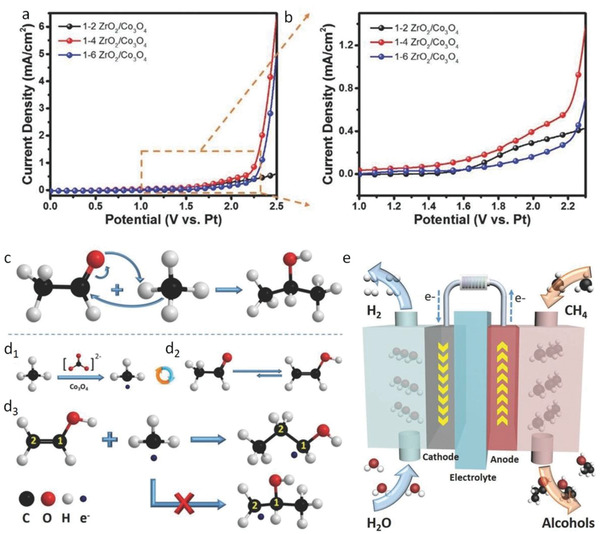
a,b) LSV curves of the ZrO_2_/Co_3_O_4_ samples with the ratios of 1:2, 1:4, and 1:6 and their magnification, respectively. c) Nucleophilic addition reaction of methane to acetaldehyde to produce 2‐propanol. d_1_–d_3_) Free radical addition reaction of methane to acetaldehyde to generate 1‐propanol. e) The electrochemical device for methane oxidation. Reproduced with permission.^[^ ^111^ ^]^ Copyright 2017, Wiley‐VCH GmbH.

Following the idea of Spinner, Park's group recently designed ZrO_2_/Co_3_O_4_ composite catalysts for the electrochemical oxidation of CH_4_ at room temperature.^[^ ^111^ ^]^ A series of composite catalysts were synthesized with different ratios of Co_3_O_4_ and ZrO_2_. Electron microscopy (**Figure** **7**) images revealed that oval‐shaped and uniform ZrO_2_ nanoparticles were anchored on the surface of Co_3_O_4_ nanoplates to make heterojunctions. Linear sweep voltammetry (LSV) curves (**Figure** **8**a,b) proved that the 1–4 ZrO_2_/Co_3_O_4_ electrocatalyst displayed the highest current density for CH_4_ oxidation. Long‐term electrochemical oxidation (at 2.0 V applied potential in a closed vessel containing 0.5 m Na_2_CO_3_, catalysts deposited on carbon paper working electrode and Pt foil counter electrode) led to the formation of 1‐propanol and 2‐propanol as the main products, with production efficiency of >60% after 12 h of oxidation. Acetaldehyde was the key intermediate from which 1‐propanol and 2‐propanol were generated (Figure 8c and d_3_). CH_3_OH, C_2_H_5_OH, CH_3_CHO, and CH_3_COCH_3_ were formed as byproducts. Notably, except methanol, all the products formed in this work were C3 species, which indicated the upgradation from an inert C1 starting material to higher‐value organics. The electrochemical device for methane oxidation is displayed in Figure 8e. Moreover, replacing Co_3_O_4_ in the previous system with a bimetallic oxide of NiCo_2_O_4_, Ma et al. described partial oxidation of CH_4_ to produce only C3 products – propionic acid, acetic acid and acetone with CH_4_ conversion efficiency of 47.5% after 20 h of reaction at room temperature.^[^ ^116^ ^]^ The selectivity for propionic acid was calculated to be ≈65% with a rate of formation of 1173 µmol g^−1^ h^−1^ after 20 h.

Recently, Song et al. engineered the interface of NiO/Ni by calcination and observed efficient electrooxidation of methane and C–C coupling in 0.1 m NaOH solution to produce ethanol under ambient conditions.^[^ ^117^ ^]^ The FE of ethanol production and yield were reported to be 89% and 25 µmol g_NiO_^−1^ h^−1^, respectively, by 3.0 NiO/Ni electrocatalyst at 1.40 V versus RHE. The catalyst maintained nearly same current density for 24 h at 1.40 V versus RHE with a slight decrease in FE for EtOH production. X‐ray photoelectron spectroscopy (XPS) analysis showed that NiO/Ni ratio was the same before and after 24 h of catalysis. Density functional theory (DFT) calculations explained highly selective ethanol formation that involved the following steps: CH_4_* → CH_3_* + H*, CH_3_* → CH_2_* + H*, CH_2_* + OH* → CH_2_OH* and CH_3_* + CH_2_OH* → CH_3_CH_2_OH*, here * denotes active site on the catalyst surface. On the other hand, Guo et al. demonstrated electrocatalytic methane oxidation over NiO@Ni hollow fiber (HF) in 0.1 m NaOH aqueous solution under ambient conditions.^[^ ^118^ ^]^ It was observed that the electrocatalyst 1%NiO@NiHF was able to achieve a FE for methanol production to 54% at 1.44 V (vs RHE), whereas the FE reached to 85% at 1.46 V for ethanol generation. The electrocatalyst was stable for 3 h.

#### 3.1.3 Electrochemical Mechanism for CH_4_ Oxidation

Selective oxidation of CH_4_ is very important for providing desired products. In many of such oxidation reactions, electrochemically activated oxygen species (O*) act as oxidizing agent. Appropriate cathodes can reductively activate O_2_ molecules to generate O* (O_2_ + 2H^+^ + 2e^−^ + M*^n^* ^+^ → O*–M*^n^* ^+^ + H_2_O, where M*^n^* ^+^ is the active site on the cathode, such as Sm^3+^, Fe^3+^, and Cu^2+^). The O* can selectively oxidize light hydrocarbons to the corresponding oxygenates (O* + R–H → R–OH). This idea was adopted by Otsuka and Yamanaka, who reported selective oxidation of CH_4_ at 28 °C on carbon whisker cathode.^[^ ^113^ ^]^ However, it was speculated that the •OH oxidized CH_4_ completely to CO_2_ at near room temperature. Later, although this idea was further improved by the same group but the O* was not potent enough to oxidize methane.^[^ ^113^, ^114^ ^]^ Nevertheless, the idea of electrochemically activated oxygen species was distinctive. This idea was further developed by Hibino and co‐workers.^[^ ^124^ ^]^ They demonstrated CH_4_ oxidation to selectively produce methanol in a hydrogen–oxygen fuel cell that contained Sn_0.9_In_0.1_P_2_O_7_ as electrolyte, which showed the capability for high proton conductivities above 80 °C, and Pd/C, Pt/C, Rh/C, Au/C, and PdAu/C (10 wt% metal basis) composites as cathodes (O_2_ + 2H^+^ + 2e^−^ → O* + H_2_O), and commercial Pt/C (60 wt% Pt) as anode (H_2_ → 2H^+^ + 2e^−^). The cathode was fed with methane and O_2_ (50 vol% each) gas mixture, whereas the anode was provided with hydrogen. Notably, small quantity of hydrogen permeated from anode, through the electrolyte, to the cathode, where it reacted with the oxygen present therein. Then, O* reacted with methane to produce methanol (O* + CH_4_ → CH_3_OH). The Pd/C catalyst was able to directly produce methanol from CH_4_, but the formation rate was not satisfactory. In contrast, the Pt/C and Au/C cathodes catalyzed the formation of CO_2_. The Rh/C cathode displayed practically insignificant catalytic activity for methane oxidation; the bimetallic Pd/Au cathode (at a ratio of 8:1) demonstrated the highest rate of methanol formation. The selectivity for methanol formation reached 60.0% at a current efficiency for CH_4_ conversion of merely 0.012% at 50 °C, with CO_2_ as the minor product. Furthermore, with the temperature increased, the selectivity toward methanol decreased, although the rate of conversion was higher. The authors speculated the possibility of electrochemical generation of H_2_O_2_ or its derivatives at the cathodes. Interested readers are suggested to go through the reference for the details.

Although these works showed a new direction for the direct and selective oxidation of CH_4_ to produce methanol at low temperatures, the current efficiency for the conversion was very low (merely 0.012%). The dominant reaction occurred at the cathode was water production (2H^+^ + 2e^−^ + 0.5O_2_ → H_2_O), instead of O* generation. This challenge was further overcome by Hibino and Lee, through carrying out CH_4_ oxidation at the anode instead of cathode in a fuel cell‐type reactor at low temperatures.^[^ ^115^ ^]^ At the anode, a mixture of CH_4_ and water vapor was supplied, whereas air was provided to the cathode. Several catalysts were used as anodes. However, a significant amount of methanol was produced on the V_2_O_5_/SnO_2_ anode using Sn_0.9_In_0.1_P_2_O_7_ proton conductor as the electrolyte at 100 °C. Unfortunately, the identification of O* was not clear to the authors. However, different reactive oxygen species O^•−^ (H_2_O → O^•−^ + 2H^+^ + e^−^) and O_2_^•−^ (2H_2_O → O_2_^•−^+ 4H^+^ + 3e^−^) were formed electrochemically over V^4+^ sites. The maximum current efficiency for methanol production was determined to be 61.4%, and the selectivity was calculated to be 88.4% at 100 °C and at ≈900 mV (measured with a Hokuto Denko HE‐101 electrometer). The reactions that occurred at the electrodes are given below

(1) $$\begin{array}{c} \mathrm{Cathode}:\;O_{2}+4H^{+}+4e^{-}\to 2H_{2}O \end{array}$$

(2) $$\begin{array}{c} \mathrm{Anode}:\;CH_{4}+H_{2}O\to CH_{3}\mathrm{OH}+2H^{+}+2e^{-} \end{array}$$

In a different approach, Ogura and Takamagari combined photochemical and electrochemical oxidation of CH_4_ at room temperature.^[^ ^109^ ^]^ A Pt plate was used as anode in a 0.6 m KCl solution of 11.0 pH and a low pressure mercury lamp (4 W) as an illuminator (254 nm). The applied potential was varied from 1.0 to 1.3 V (vs SCE). Depending on the potential applied, the products generated were methyl chloride, methanol, methylene dichloride and a small amount of trichloromethane. Following the same approach, this group further reported the realization of methanol and methyl chloride from CH_4_ at room temperature.^[^ ^110^ ^]^ The process involved was electrochemical oxidation of chloride ions to chlorine molecules, followed by the generation of chloride radicals upon light illumination, which triggered the activation of CH_4_. The main advantage of these works is that CH_4_ was directly oxidized to methanol without further oxidation.

#### 3.1.4 The Shilov Cycle

**Scheme 1:**
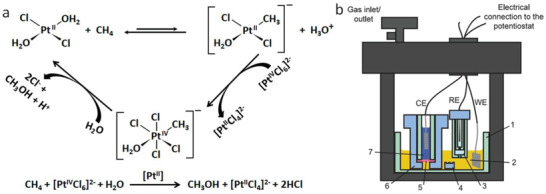
a) Shilov cycle and overall chemical reaction for electrochemical selective oxidation of methane to methanol. b) The diagram of the electrochemical cell used for methane oxidation by Surendranath et al. WE: working electrode, Pt foil; RE: reference electrode, Ag/AgCl; CE: counter electrode, Pt mesh. 1: glass cell; 2: reaction solution containing Pt salt; 3: fritted tube for making room of RE; 4: PTFE stir bar; 5: H^+^ ion conducting membrane; 6: PTFE body holding the membrane stack and 7: counter electrode compartment containing sacrificial electron acceptor VOSO_4_ (3 m). The WE compartment contained 3 × 10^−3^ m K_2_PtCl_4_, 7 × 10^−3^ m Na_2_PtCl_6_⋅6H_2_O, 10 × 10^−3^ m NaCl, and 0.5 m H_2_SO_4_. The cell and solution were O_2_‐free and pressurized with CH_4_ at 500 psi and the temperature was 130 °C. Reproduced with permission.^[^ ^130^ ^]^ Copyright 2019, American Chemical Society (https://pubs.acs.org/doi/10.1021/acscentsci.9b00273, further permission related to the material excerpted should be directed to the ACS).

Another way to realize CH_4_ oxidation is to use the Shilov cycle named after Alexander E. Shilov.^[^ ^125^, ^126^, ^127^ ^]^ We will mention it here briefly for academic interest. The Shilov system is a classic example of C—H bond activation in which stronger C—H bonds preferentially undergo partial oxidation to the respective alcohol over the weaker ones. It is catalyzed by PtCl_2_ in an aqueous solution, where PtCl_6_^2−^ acts as the ultimate oxidizing agent. In the first step, electrophilic addition of CH_4_ (or an alkane) to the Pt(II) center of chloroplatinate occurs in an aqueous medium. Simultaneous deprotonation leads to the formation of a Pt(II)–CH_3_ complex. In the second step, [Pt(IV)Cl_6_]^2−^ oxidizes the Pt(II)–CH_3_ complex to form the Pt(IV)–CH_3_ complex. Subsequent nucleophilic attack by H_2_O molecules or Cl^−^ ions at the methyl group of the Pt(IV)–CH_3_ complex leads to the formation of methanol or methyl chloride (which is easily hydrolyzable to methanol), with simultaneous regeneration of the Pt(II).^[^ ^128^ ^]^ A schematic of the Shilov cycle and the overall reaction are shown in **Scheme** **1**a.

However, there is no practical application for CH_4_ oxidation by this cycle. Fortunately, with water soluble *p*‐toluenesulfonic acid (*p*‐TsOH, 0.21 m) as a model substrate for CH_4_ electrochemical oxidation was reported,^[^ ^129^ ^]^ Liu and Nusrat realized electrocatalytic conversion of *p*‐TsOH as a surrogate of CH_4_ in 0.5 m H_2_SO_4_ at about 560 mV (vs Ag/AgCl) applied potential with a homogeneous solution of Na_2_PtCl_4_ as a Pt(II) catalyst,^[^ ^128^ ^]^ expecting similar catalyst process for electrocatalytic CH_4_ oxidation to methanol. Notably, the originally reported Shilov cycle suffers from the requirements of stoichiometric Pt(IV) that is not economically viable. In this regard, recently Kim and Surendranath reported continuous regeneration of Pt(IV) through the electrochemical route.^[^ ^130^ ^]^ It was noted that the metallic Pt electrode (with surface adsorbed Cl^−^ ions) drove oxidation of Pt(II) through an inner sphere electron transfer mechanism (that involved transfer of the surface adsorbed Cl to Pt(II)) to form Pt(IV), at low overpotential. Thus by monitoring the potential of the Pt(II) catalyzed methane oxidation, the Pt(II)/Pt(IV) ratio was maintained resulting in continuous formation of methanol. The catalyst system was able to produce methanol for 30 h with selectivity for CH_3_OH and CH_3_Cl production of >80% (70% selectivity for CH_3_OH). It should be mentioned here that further oxidation of MeOH was suppressed by the presence of excess Cl ions (from NaCl) that also maintained the Pt(II)/Pt(IV) concentration ratio. The methane oxidation reaction was performed at >100 °C in a home‐built high‐pressure two compartment electrochemical cell shown in the diagram in Scheme 1b.

### 3.2 Photocatalytic Oxidation of CH_4_

**Figure 9:**
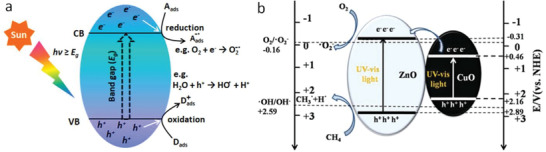
Generalized photocatalytic scheme a), *A*_ads_ and *D*_ads_ are, respectively, the adsorbed acceptor and adsorbed donor species over the photocatalyst. b) Bandgap positions of a photocatalyst to carry out CH_4_ oxidation with example of ZnO and CuO. Reproduced with permission.^[^ ^143^ ^]^ Copyright 2019, The Royal Society of Chemistry.

Photocatalysis is a widely studied technique for organic dye degradation and water purification including water decontamination and disinfection.^[^ ^131^, ^132^ ^]^ In a typical photocatalytic process, a semiconductor is excited with a light source to generate electron–hole pairs. The resultant electrons and holes possess kinetic energy equal to the bandgap value of the semiconductor. The electrons transport to the conduction band and perform reduction, whereas the holes occupy the valence band and execute oxidation.^[^ ^133^, ^134^ ^]^ A generalized photocatalytic process is depicted in **Figure** **9**a. Photocatalysis enables difficult chemical reactions to occur at room/low temperatures. Therefore, this process offers low cost oxidation of methane and enhanced catalyst stability. In contrary, thermal and enzymatic methane oxidation confront several challenges as discussed in Section 3.1. For the photocatalytic oxidation of methane to occur, suitable photocatalysts are essential, in order to generate highly energetic oxygen species, such as O_2_^−^ and •OH radicals. Therefore, a photocatalyst must have a conduction band minimum more negative than the potential of the O_2_/O_2_^−^ redox couple (−0.16 V vs normal hydrogen electrode, NHE) and a valence band maximum more positive than the potential of the •OH/OH^−^ redox couple (+2.59 V vs NHE) (Figure 9b).^[^ ^135^, ^136^, ^137^ ^]^ As we mentioned earlier, we are not going to discuss the photocatalytic conversion of CH_4_ to higher alkanes. Interested readers are referred to the reviews.^[^ ^21^ ^]^ Numerous photocatalysts have been investigated for CH_4_ oxidation.^[^ ^138^ ^]^ In the following sections, we discuss different processes for the photocatalytic CH_4_ oxidation at low temperatures, summarized in **Table** **3**.

**Table 3:** Summaries of low temperature photocatalytic processes for CH_4_ oxidation

Catalyst	*T* [°C]; *P* [Pa]	Light	Oxidant	Product [s]	Ref.
V/SiO_2_, TiO_2_, P/SiO_2_	RT	UV, *γ*	O_2_	CO_2_	^[^ ^139^ ^]^
Cu‐doped MoO_3_	100	UV	O_2_	MeOH	^[^ ^140^ ^]^
TiO_2_, TiO_2_/MoO_3_, TiO_2_/MoO_3_/H_4_SiW_12_O_40_	RT	Solar simulator	O_2_	CO_2_, CO	^[^ ^141^ ^]^
TiO_2_/H_4_SiW_12_O_40_	RT	Solar simulator	O_2_	CO, CO_2_, H_2_O	^[^ ^142^ ^]^
UO_2_^2+^/MCM‐41	RT	Sun	O_2_	CO_2_	^[^ ^161^ ^]^
ZnO and Ag/ZnO	RT	300 W Xe lamp	O_2_	CO_2_	^[^ ^136^ ^]^
CuO/ZnO	RT	Xe lamp	O_2_	CO_2_	^[^ ^143^ ^]^
Cu, La, Pt, Cu/La‐doped WO_3_	98/10.1 m	UV/visible	H_2_O	MeOH, H_2_	^[^ ^144^ ^]^
Beta zeolites	RT	Deep UV	O_2_	MeOH	^[^ ^145^ ^]^
WO_3_	RT	Visible laser	H_2_O_2_	MeOH, CO_2_, O_2_	^[^ ^146^ ^]^
Ag^+^ impregnated WO_3_	RT	355 nm laser	H_2_O	MeOH	^[^ ^147^ ^]^
Mesoporous WO_3_	55	Hg vapor lamp	H_2_O	MeOH	^[^ ^148^ ^]^
La‐doped mesoporous WO_3_	55	UVC–visible	H_2_O	MeOH	^[^ ^149^ ^]^
MMT‐modified TiO_2_ ^a)^	100	Hg lamp	CO_2_	CO, MeOH	^[^ ^150^ ^]^
CuPc‐modified TiO_2_ ^b)^	RT	Visible light	CO_2_	CO, AcOH, CH_3_CHO	^[^ ^151^ ^]^
ZnS–ZnO	RT	UV–visible	CO_2_	–	^[^ ^152^ ^]^
Co‐doped Al_2_O_3_/Co nanoparticles	RT	UV–visible–IR	CO_2_	CO, H_2_	^[^ ^153^ ^]^
Photochemical oxidation	<100	20 W low pressure Hg lamp	Water vapor	MeOH, AcOH, HCOOH, EtOH, acetone	^[^ ^76^ ^]^
Ru single atoms on Cu nanoparticles	RT	19.2 W cm^−2^ white light illuminator	CO_2_	CO and H_2_	^[^ ^92^ ^]^
ZnO/La_0.8_Sr_0.2_CoO_3_	RT	Solar light	O_2_	CO_2_ and H_2_O	^[^ ^154^ ^]^
FeOOH/m‐WO_3_	RT	Visible light	H_2_O_2_	MeOH	^[^ ^155^ ^]^
Cocatalyst (Pt, Pd, Au or Ag)/ZnO	RT	Solar light	O_2_	MeOH and HCHO	^[^ ^156^ ^]^
Rh/SrTiO_3_ ^c)^	RT	UV light	CO_2_	CO and H_2_	^[^ ^157^ ^]^
BiVO_4_ microcrystals	65	350 W Xe lamp	H_2_O	MeOH	^[^ ^22^ ^]^
0.33 metal wt% FeO*_x_*/TiO_2_	25	300 W Xe lamp	H_2_O_2_	MeOH	^[^ ^158^ ^]^

^a)^ Montmorillonite;

^b)^ Phthalocyanine;

^c)^ Although the experiment was carried out at RT, the temperature of the catalyst reached to 300 °C, we mentioned this work as the temperature was generated from the irradiation, i.e., not applied from outside.

#### 3.2.1 Photocatalytic Conversion of CH_4_ with O_2_

Recently, photocatalytic partial oxidation of CH_4_ by oxygen to obtain products, such as methanol, formaldehyde, and CO, has been an interesting topic of research. However, the oxidation of CH_4_ by O_2_ is a spin‐forbidden process because CH_4_ and its products (such as methanol) are in the singlet (ground) state, whereas oxygen is in the triplet electronic state.^[^ ^138^ ^]^

In 1978, for the first time, CH_4_ was photocatalytically converted by oxygen under UV light exposure on V/SiO_2_ and TiO_2_ and *γ* irradiation on V/SiO_2_ and P/SiO_2_ photocatalysts.^[^ ^139^ ^]^ In each case, upon light exposure hole centers, O^−^ species were generated (O^2−^ + h^+^ → O^−^), which attacked CH_4_ to yield methyl radicals that ultimately formed methoxide (CH_3_O^−^). Besides, CO, C_2_H_6_, and a trace amount of CO_2_ were produced (in the absence of O_2_ and at up to 88 °C) on *γ* irradiated V/SiO_2_. Unfortunately, hazardous *γ* radiation was employed in this work. For further improvement, using V_2_O_5_ (V_2_O_5_/SiO_2_‐IW (incipient wetness), 0.6 mol% V) instead of V led to the photooxidation of CH_4_ to produce formaldehyde (corresponding to 76 mol% selectivity and 0.48 mol% one‐pass yield) under UV radiation (<310 nm) at 120 °C after 2 h of reaction.^[^ ^159^ ^]^ Both UV light and a temperature were essential for this reaction. A tetravalent vanadium (with V = O) was excited by UV light forming a charge–transfer complex, wherein a positive hole was confined by an oxygen atom to produce a strongly electrophilic O^−^ radical ion species. CH_4_ was adsorbed on the photoactivated species, which would activate the C—H bonds. Molecular O_2_ could be adsorbed at the electron rich site of the generated intermediate, which could abstract hydrogen to produce HCHO. O^−^ Centers were created by the charge–transfer excitation form O 2p to the metal valence d orbitals. The lifetime of the photogenerated electron–hole pairs was the decider for the activity of the O^−^ centers. The longer lifetime of the excited state provides more efficient CH_4_ photoexcitation by the O^−^ centers. For example, in MoO_3_, the lifetime of the electron–hole excited state was determined to be 63 µs, which could be increased by Cu doping.^[^ ^160^ ^]^ Through theoretical, as well as, experimental studies, Ward et al. proved that Cu‐doped MoO_3_ photocatalysts are more effective for heterolytic C—H bond activation than pristine MoO_3_.^[^ ^140^ ^]^ In this process, CH_4_ was partially oxidized to CH_3_OH in the presence of O_2_ at 100 °C. Methanol formation rate was the maximum when the concentrations of Cu and Mo were equal. The Cu doping makes MoO_3_ (resulting in CuMoO_4_) visible light active due to the presence of empty and filled orbitals (Cu 3d and O 2p) in the bandgap region between the O 2p and Mo 4d orbitals. Thus, some holes were stabilized by the alternative pathway, resulting in an increased lifetime of the excited state and the O^−^ sites.

In a further improvement, Grätzel and co‐workers reported activation of CH_4_ by O_2_ on different photocatalysts illuminated by a solar simulator at room temperature and atmospheric pressure.^[^ ^141^ ^]^ Pure TiO_2_ led to the formation of CO_2_. However, deposition of 4% MoO_3_ on TiO_2_ produced a mixture product of CO and CO_2_. Interestingly, when TiO_2_ was loaded with both MoO_3_ and H_4_SiW_12_O_40_, CO was the main product. Contrarily, only tungstosilicate (SiW_12_O_40_)^4−^‐loaded TiO_2_ activated CH_4_ to produce CO, CO_2_, and H_2_O.^[^ ^142^ ^]^ To carry out photocatalysis in practical, sunlight is more desirable than solar simulator. In this endeavor, Krishna et al. used sunlight for room temperature photooxidation of CH_4_ in air by uranyl ions anchored within the mesopores of MCM‐41 silicate.^[^ ^161^ ^]^ The UO_2_^2+^ ions were tightly attached to the silicate and caused the latter to absorb visible light. CH_4_ was converted 100% within 2.5 h. However, due to the long life and strongly oxidizing oxidation state of uranyl ions, CO_2_ was formed selectively.

**Figure 10:**
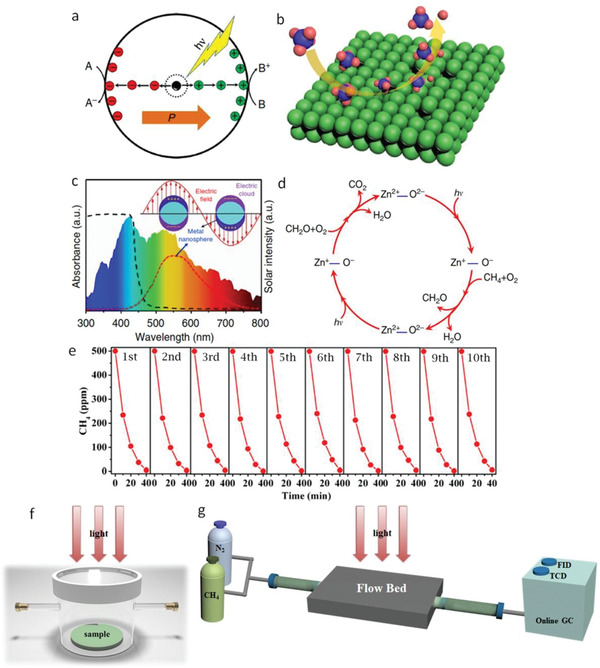
a) Polarity on photocatalyst induces fast charge separation and transport. b) Surface defects on photocatalyst leads to faster surface reaction. c) Surface decorated metal nanoparticles increases absorption of solar spectrum. d) Mechanism of photooxidation of methane. Stability of the catalyst for ten cycles. e) Schematic diagrams of photocatalytic instruments a) fixed‐bed and b) flow‐bed reactor that were used for photocatalytic methane conversion.^[^ ^143^, ^163^, ^162^ ^]^ GC, FID, and TCD are gas chromatograph, flame ionization detector, and thermal conductivity detector, respectively. Reproduced with permission.^[^ ^162^ ^]^ Copyright 2016, Springer Nature.

An efficient photocatalyst would absorb the visible region (preferably) of the solar spectrum to generate more photogenerated charge carriers.^[^ ^133^ ^]^ The loading of metal nanoparticles (especially noble metals) on the main photocatalysts often increases light absorption.^[^ ^134^ ^]^ Recently, Chen et al. demonstrated high CH_4_ oxidation activity of ZnO nanoparticles loaded with Ag nanoparticles under simulated sunlight.^[^ ^162^ ^]^ The deposited Ag nanoparticles showed surface plasmon resonance and reduced recombination rate of the photogenerated charge carriers (**Figure** **10**a), with faster surface reaction (Figure 10b) and increased visible light absorption (Figure 10c). A two‐step mechanism for CH_4_ oxidation was proposed (Figure 10d). In the first step, CH_4_ reacted with oxygen to produce H_2_O and HCHO (CH_4_ + O_2_ → HCHO + H_2_O). HCHO, as the intermediate, reacted further with O_2_ to produce CO_2_ and H_2_O in the second step (HCHO + O_2_ → CO_2_ + H_2_O). The quantum yield of 8% was obtained at <400 nm wavelengths. The photocatalytic performance and photocatalyst remained unchanged after ten cycles, as shown in Figure 10e in a fixed‐bed reactor revealed by the characterizations of X‐ray diffraction (XRD), XPS, and optical absorption measurements. Moreover, it maintained its catalytic activity for 50 h in a flow‐gas mode. Thus the photocatalyst was very stable for methane oxidation. Photocatalytic instruments in a fixed‐bed reactor and flow‐gas mode are represented in Figure 10f,g. For further development, Ag nanoparticles were replaced by inexpensive CuO nanoparticles (<1 wt%), which resulted in more efficient CH_4_ oxidation under ambient conditions.^[^ ^143^ ^]^

Notably, the band edge potential of CuO did not permit it to activate oxygen molecules (Figure 9b). Therefore, after excited to the conduction band of CuO, a fraction of the enriched electrons could be further excited to the conduction band of ZnO nanoparticles, satisfying sufficient energy to activate oxygen molecules (Figure 9b). The photoactivity of the catalyst system was very weak under the visible light illumination, as the electron transfer from CuO to ZnO was not easy under this condition. Nevertheless, this catalyst showed excellent stability for over a period of 450 min. Recently, Yang et al. demonstrated solar driven photocatalytic oxidation of methane at the epitaxial heterointerface of ZnO/La_0.8_Sr_0.2_CoO_3_ to produce CO_2_.^[^ ^155^ ^]^ The photothermal effect of the solar illumination boosted methane oxidation by ≈2 times by enhancing the electron transfer at the interface. Although these reports show considerable progress for CH_4_ oxidation by molecular O_2_, technologically unimportant CO_2_ was often generated. Achieving high catalytic activity and selectivity remained challenging, especially for partial oxidation products.

Nevertheless, co‐catalyst (Pt, Pd, Au, or Ag) loaded ZnO oxidized CH_4_ selectively to CH_3_OH and HCHO in the presence of molecular O_2_ at room temperature in aqueous medium as demonstrated by Song et al.^[^ ^156^ ^]^ The photogenerated holes and electrons of the catalyst system activated CH_4_ and molecular O_2_ into •CH_3_ and •OOH radicals, respectively, which subsequently converted to oxygenates. Notably, the mild reactive •OOH radicals stopped further oxidation of the oxygenates. Up to 250 µmol oxygenates, with ≈95% selectivity, were generated by 0.1 wt% Au loaded ZnO after 2 h of photocatalytic experiment. It should be pointed out here that compared to molecular O_2_; nitric oxide (NO) is a milder oxidizing agent that can oxidize CH_4_ selectively to MeOH by a photocatalyst at room temperature.^[^ ^163^ ^]^

#### 3.2.2 Photocatalytic Conversion of CH_4_ with Water

Direct conversion of CH_4_ to oxygenates in the presence of steam (photocatalytic steam reforming of methane or SRM) via photocatalytic processes is of great significance. This conversion proceeds according to the following reaction:

(3) $$\begin{array}{c} CH_{4}+H_{2}O\to CH_{3}\mathrm{OH}+H_{2}\;\Delta G_{298K}=117\;\mathrm{kJ}\;\mathrm{mo}l^{-1} \end{array}$$

Initial experiments of such process were carried out at the National Energy Technology Laboratory (Department of Energy, United States).^[^ ^144^, ^164^ ^]^ Taylor and co‐workers converted CH_4_ to methanol, hydrogen and acetic acid overdoped WO_3_ (dopants were Cu, La, Pt and a mixture of Cu and La) photocatalysts at a temperature of ≈98 °C under atmospheric pressure.^[^ ^164^, ^165^ ^]^ Methyl viologen dichloride (1,1′‐dimethyl‐4,4′‐bipyridinium dichloride, MV^2+^) hydrate was used as the electron transferring agent. Photolysis of water, overdoped WO_3_ in the presence of MV^2+^, first generated hydroxyl radicals, which abstracted a hydrogen atom from CH_4_ to produce methyl radicals. Successively, the •CH_3_ radicals reacted with water to form CH_3_OH. Meanwhile, photocatalytic conversion of CH_4_ dissolved in water and methane hydrate overdoped WO_3_ and TiO_2_ photocatalysts in the presence of MV^2+^ was investigated.^[^ ^144^ ^]^ The photoconversion of CH_4_ and methane hydrate was not successful below 70 °C and 1.0 MPa. However, conversion occurred at 50 °C under 10.1 MPa. Contrarily, methane hydrate underwent conversion at temperatures as low as −15 °C. The products did not depend on the pressure. It was further observed that CH_4_ conversion and methanol production increased with the addition of H_2_O_2_, suggesting involvement of hydroxyl radicals as an intermediate. La‐doped WO_3_ showed the highest CH_4_ conversion efficiency. The main products were methanol and hydrogen. The general reaction pathway for these two works is given below. However, the generated methanol could possibly combine with holes to produce formic acid, carbon monoxide and carbon dioxide. This phenomenon indicated a selectivity challenge for this CH_4_ activation process. Moreover, the electron transfer agent, methyl viologen is a moderately expensive chemical.

(4) $$\begin{array}{c} \mathrm{La}/WO_{3}\overset{hv}{\underset{\lambda \;\geq \;410\;\mathrm{nm}}{\to }}e_{\mathrm{CB}}^{-}+h_{\mathrm{VB}}^{+} \end{array}$$

(5) $$\begin{array}{c} e_{\mathrm{CB}}^{-}+MV^{2+}\to MV^{•+} \end{array}$$

(6) $$\begin{array}{c} h_{\mathrm{VB}}^{+}+H_{2}O\to H^{+}+•\mathrm{OH} \end{array}$$

(7) $$\begin{array}{c} MV^{•+}+H^{+}\to \frac{1}{2}H_{2}+MV^{2+} \end{array}$$

(8) $$\begin{array}{c} CH_{4}+•\mathrm{OH}\to •CH_{3}+H_{2}O \end{array}$$

(9) $$\begin{array}{c} •CH_{3}+H_{2}O\to CH_{3}\mathrm{OH}+\frac{1}{2}H_{2} \end{array}$$

Here, *e* ^−^_CB_ and *h* ^+^_VB_ represent electrons in the conduction band and holes in the valence band, respectively.

**Figure 11:**
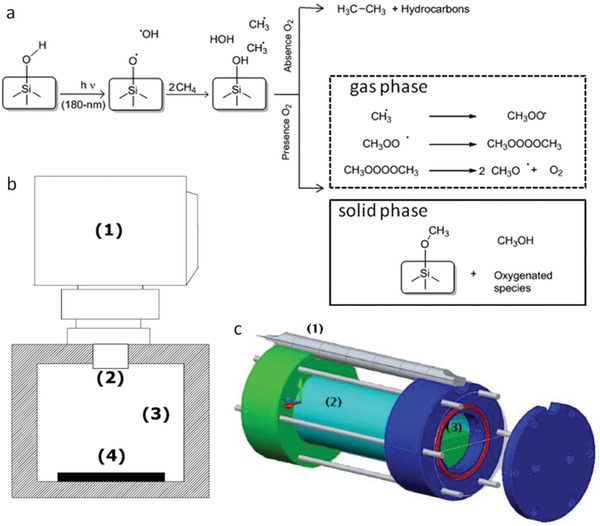
a) Schematic representation of CH_4_ oxidation on a silica surface under deep UV light. b) 165 nm photoreactor consisting of 1. deuterium lamp, 2. MgF2 window, 3. gas chamber, and 4. photocatalyst. c) 185 nm photoreactor, 1. Hg lamp, 2. synthetic quartz window, and 3. gas chamber, wherein photocatalyst was placed. Reproduced with permission.^[^ ^145^ ^]^ Copyright 2011, American Chemical Society.

Interestingly, in the above works, methane hydrate was used with some special advantages. It contained much higher concentration of methane (15 mol%) than that can be obtained in a pressurized reactor containing CH_4_ and H_2_O. Besides, methane hydrate provided the restricted mobility and close proximity of the •OH radicals with CH_4_ molecules. In a dramatic improvement, room temperature transformation of CH_4_ into C1 oxygenates was carried out by Garcia's group under deep (<200 nm) UV illumination in the presence of water and air over the confined space of zeolites’ solid surfaces (e.g., beta zeolites) containing hydroxyl functionality (i.e., internal silanol).^[^ ^145^ ^]^ The OH groups on zeolites were cleaved homolytically under deep UV radiation (165 and 185 nm) and generated surface siloxyl (hydroxyl in case of water) radicals in the confined micropores of the zeolites (**Figure** **11**a). In the micropores, •OH radicals scavenged the initially formed methyl radicals. As a result, •OH radicals were not accessible to large amount of methyl radicals, diminishing the side reactions significantly. Therefore, selectivity toward C1 oxygenate was over 95% at a conversion of 13% within a few minutes. In addition, the authors demonstrated that 7.16 Gcal mol^−1^ energy was required for 13% CH_4_ conversion (185 nm lamp, 1 h irradiation) against 15.9 Gcal mol^−1^ energy for CH_4_ transformation to syn gas. However, in the absence of oxygen, low molecular weight alkanes were produced.^[^ ^166^ ^]^ The proposed mechanism for the transformation of CH_4_ using deep UV light over a silica surface is shown in Figure 11a. The selectivity toward methanol was excellent, although hazardous deep UV radiation was used as light source. Two types of UV photoreactors were used for methane conversion as shown in Figure 11b,c.

It is interesting to use laser as light source in photocatalysis because of its high intensity, monochromaticity, and tunability. In this endeavor, Gondal et al. described photocatalytic CH_4_ conversion to generate methanol using a visible laser (argon ion laser, 514 nm) beam over WO_3_.^[^ ^146^ ^]^ The conversion rate was much faster (within 15 min) compared to the conventional lamps (≈18 h) and the products were analyzed to be methanol, O_2_ and CO_2_. High photon density of laser generated very large concentration of •OH radicals, which combined to produce H_2_O_2_ as main source of O_2_. However, visible laser can show photoactivity only on photocatalysts with bandgap ≤≈3 eV. Therefore, the same group investigated UV laser beam (355 nm)‐induced photocatalytic CH_4_ conversion over WO_3_, rutile TiO_2_, and NiO photocatalysts at room temperature in aqueous suspensions.^[^ ^167^ ^]^ A maximum conversion of ≈29% occurred with WO_3_ photocatalyst for methanol production. In these works, CH_4_ conversion efficiency was not sufficient due to the recombination of the photogenerated charge carriers. This issue was resolved by using Ag^+^ impregnated WO_3_, wherein the former suppressed the charge recombination rate appreciably to generate higher concentration of •OH under a laser beam (100 mJ, 355 nm).^[^ ^147^ ^]^ CH_4_ was converted to methanol with simultaneous formation of hydrogen and oxygen. However, due to the high reactivity of the hydroxyl radicals, several complex side reactions occurred during the photocatalytic process. Moreover, the electron donating nature of methanol limited the yield and selectivity of the product.

**Figure 12:**
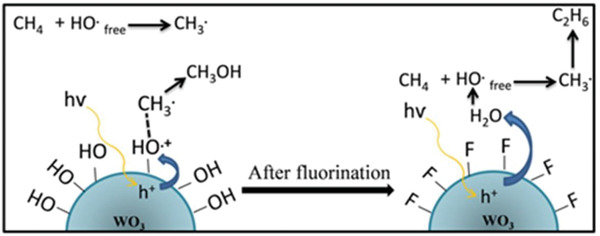
Proposed mechanism for selective oxidation of CH_4_ over tungsten oxide and fluorine‐modified tungsten oxide. Reproduced with permission.^[^ ^168^ ^]^ Copyright 2014, Elsevier.

Above all, WO_3_‐based photocatalysts are attractive for CH_4_ conversion due to the energy of its valence band maxima (*E*_VB_ = +3.1 V vs NHE), which is suitable for water oxidation to generate hydroxyl radicals successively producing methanol.^[^ ^146^ ^]^ Nanostructured, such as ordered mesoporous, WO_3_ could have enhanced photocatalytic effect due to its porous structure and high specific surface area, which ensure more active sites. In this regard, the work of Villa et al. was noteworthy, who studied the photocatalytic conversion of CH_4_ to methanol in an aqueous suspension over ordered mesoporous WO_3_ exposed to a medium pressure quartz mercury vapor lamp at 55 °C.^[^ ^148^ ^]^ The authors also experimented to understand the effect of the addition of H_2_O_2_ and electron scavengers, such as Fe^3+^, Cu^2+^ and Ag^+^. Methanol production was improved significantly in the presence of Fe^3+^ and Cu^2+^ ions, as these species scavenged photogenerated electrons leading to better charge separation. An impressive yield of 55.5 µmol h^−1^ g^−1^ was obtained with Fe^3+^ (2 × 10^−3^ m) incorporated mesoporous WO_3_. In a contrary, in the presence of Ag^+^ ions, methanol production decreased compared to the pristine mesoporous WO_3_. The negative effect of Ag^+^ ions was attributed to its reduction and successive deposition of the resulting metallic Ag on WO_3_. Surface modification of WO_3_ with fluorine decreased the yield of methanol production,^[^ ^168^ ^]^ which could be explained by the generation/presence of excess amount of hydroxyl radicals that favored the formation of ethane. The mechanism of CH_4_ oxidation on WO_3_ and surface modified WO_3_ with fluorine are shown in **Figure** **12**. This issue of lower amount of methanol production was addressed by La doping into mesoporous WO_3_. A twofold higher product formation rate for methanol compared to pristine WO_3_ was observed, while the CO_2_ generation rate was decreased.^[^ ^149^ ^]^ Considering the importance of developing a photocatalytic system for the direct conversion of CH_4_ to methanol, only limited efforts have been made in this direction to date. Thus, the advancement of such systems does not meet the demand expected for both academic interests and industrial applications.

**Figure 13:**
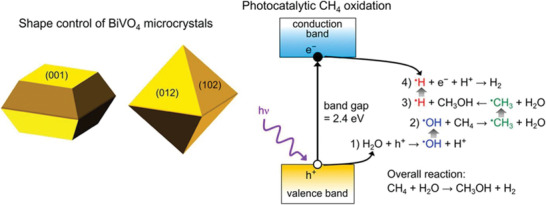
Facet‐dependent conversion of CH_4_ to MeOH over BiVO_4_ microcrystals and mechanism of the photocatalysis. Reproduced with permission.^[^ ^22^ ^]^ Copyright 2018, American Chemical Society.

Exposed catalyst surface can have significant effect on CH_4_ oxidation activity as well as product selectivity as demonstrated by Zhu et al.^[^ ^22^ ^]^ It was noted that the (102) and (012) facets of bipyramidal BiVO_4_ microcrystals were more active and more selective for MeOH production compared to the (001) facets of platelet microcrystals (**Figure** **13**). The highest activity for MeOH production was 151.7 µmol h^−1^ g^−1^ with > 85% selectivity for 2 h of photocatalysis. The catalytic activity was maintained for >100 µmol h^−1^ g^−1^ for up to 5 h of photocatalysis. However, thin platelets produced CO_2_ as the major product. The valence band‐edge of BiVO_4_ possesses sufficient energy and the photogenerated holes therein can generate •OH radicals from water oxidation. The successive reactions are shown in Figure 13.

#### 3.2.3 Photocatalytic Conversion of CH_4_ with CO_2_

In this process, also known as photocatalytic dry reforming of methane (DRM), CO_2_ is used to oxidize CH_4_, showing high endothermic nature of the overall chemical reaction (CO_2_ + CH_4_ → 2CO + 2H_2_, Δ*H*_298 K_ = 247 kJ mol^−1^).^[^ ^169^, ^170^, ^171^ ^]^ The products are not only two important fuels but also vital feedstock for synthesizing valuable chemicals. Moreover, this process minimizes the emission of two greenhouse gases. But CO_2_ is more stable (O=C=O, 532 kJ mol^−1^ at 298 K) than CH_4_ and activation of the C=O bonds is more difficult. Therefore, the conversion of CH_4_ and CO_2_ to other molecules is thermodynamically unfavorable (Δ*G*_298 K_ = 170 kJ mol^−1^). Usually, high temperatures are necessary to carry out such conversion. Fortunately, in the presence of photoenergy, a photocatalyst can initiate the process at low temperatures.

In such conversion process, usually CO forms from the reduction of CO_2_ by CH_4_.^[^ ^150^ ^]^ Therefore, it is actually reduction of CO_2_ rather than oxidation of CH_4_. Nevertheless, Yazdanpour and Sharifnia described the photoconversion of CH_4_ and CO_2_ over a copper phthalocyanine (CuPc)‐modified TiO_2_ (CuPc/TiO_2_)‐coated stainless steel photocatalyst in a gas phase batch reactor.^[^ ^151^ ^]^ The importance of this work lied in its operation under visible light. Approximately 14% and 18% conversion of, respectively, CO_2_ and CH_4_ were observed after 240 h. The formed products were different oxygenates, such as CO, aldehyde and ketone. The presence of CuPc shifted the band edge of TiO_2_ to the visible region, causing the conversion process to occur under visible light. In order to increase the conversion efficiency, reduction of CO_2_ with CH_4_ over ZnS/ZnO nanocomposites under UV and visible light was studied. ≈45% and 54% of CO_2_ and CH_4_ were converted after 5 h of UV light exposure.^[^ ^152^ ^]^

**Figure 14:**
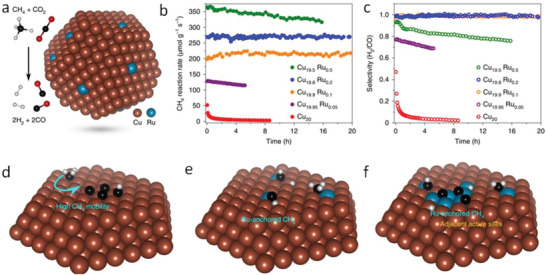
a) Ru single atoms on Cu nanoparticles and the chemical reaction of dry reforming of methane. b) Reaction rate and stability of different catalysts with variable amounts of Ru concentration and c) their selectivity. Coke resistance of different catalysts d) pure Cu nanoparticles that are susceptible to form coke from dehydrogenated CH_4_ (CH*_x_*), e) Ru single atoms (Cu_19.9_Ru_0.1_ and Cu_19.8_Ru_0.2_) suppressed coking by isolating the CH*_x_* intermediates, and f) high Ru concentration that produced coke. Reproduced with permission.^[^ ^92^ ^]^ Copyright 2020, Springer Nature.

In a significant progress, Zhou et al. reported photocatalytic DRM over Ru single atoms supported on plasmonic Cu nanoparticles (**Figure** **14**a).^[^ ^92^ ^]^ The plasmonic Cu nanoparticles enabled the alloy to absorb light strongly, while the Ru single atoms increased the catalytic activity. It was noticed that the concentration of Ru single atoms significantly influenced the reaction rate and stability of the catalyst. Pure Cu nanoparticles showed an initial reaction rate of ≈50 µmol CH_4_ g^−1^ s^−1^, which decreased to ≈4 µmol CH_4_ g^−1^ s^−1^ after ≈5 h (Figure 14b) due to the coke deposition around the catalyst (Figure 14d,f). Addition of extremely low amount of Ru (Cu_19.95_Ru_0.05_) greatly increased the initial reaction rate and stability. Further increase of Ru concentration (Cu_19.9_Ru_0.1_ and Cu_19.8_Ru_0.2_) led to achieve stability for 20 h (Figure 14c,e) after which the morphology and the chemical state of the catalyst remained intact. The stability was maintained for 50 h with a minimum loss of catalytic activity for Cu_19.9_Ru_0.1_. At the same time, selectivity (ratio of H_2_ to CO production rate) of Cu_19.9_Ru_0.1_ and Cu_19.8_Ru_0.2_ approached the ideal value 1 (i.e., >99% selectivity). Recently Shoji et al. reported DRM over Rh/SrTiO_3_ (STO) under UV light irradiation.^[^ ^157^ ^]^ The Rh nanoparticles acted as the catalytic centers. The catalyst achieved >50% DRM conversion. Although heat was not applied from outside, the catalyst was heated up to 300 °C from the UV irradiation. Notably, in photothermal catalysis the surface temperature can reach 300–500 °C or more and we are not going to discuss it further.^[^ ^172^ ^]^

#### 3.2.4 Photocatalytic Conversion of CH_4_ with NH_3_

As this strategy is not well established and the literature is not rich, we will discuss it briefly. Amino acids and other important molecules were experimentally proved to be produced from a variety of reducing gases, such as CH_4_, H_2_S, and NH_3_ using a range of energy sources, such as UV light, spark discharge, thermal energy, shock waves, and ionizing radiation.^[^ ^173^, ^174^, ^175^, ^176^, ^177^ ^]^ Keeping it in mind, Sagan and Khare reported synthesis of amino acids from a reducing gas mixture of CH_4_, H_2_S, NH_3_, and H_2_O under UV irradiation.^[^ ^178^ ^]^ H_2_S was the long‐wavelength photon acceptor for such prebiological organic synthesis. Later, Reiche and Bard reported the production of amino acids by photosynthesis from a mixture of CH_4_, NH_3_, and H_2_O over a Pt/TiO_2_ photocatalyst.^[^ ^179^ ^]^ Irradiation of a deaerated aqueous solution of NH_4_Cl (2 m) or NH_3_ (≈28%) under continuous and slow CH_4_ bubbling in the presence of Pt/TiO_2_ (platinized) illuminated by a xenon lamp (2.5 kW operated at 1.6 kW) produced a mixture of amino acids after 64–66 h. ≈0.5 and 0.2 µmol amino acids were produced in an NH_4_Cl and NH_3_ solution, respectively. The amino acids glycine, alanine, serine, aspartic acid, and glutamic acid were generated. This result demonstrated the photosynthesis of amino acids in a heterogeneous system under irradiation by near‐UV–visible light. The photosynthesis reaction did not occur on the pure TiO_2_ surface due to the lack of reduction sites. The overall reaction was as follows

(10) $$\begin{array}{ccc} & & 2CH_{4}+NH_{3}+2H_{2}O\to NH_{2}CH_{2}\mathrm{COOH}+5H_{2}\;\Delta G{}^{\circ }\\ & & \;=231.7\;\mathrm{kJ}\;\mathrm{mo}l^{-1} \end{array}$$

Mechanism studies by Bard and co‐workers revealed the involvement of hydroxyl radicals in the photosynthesis process.^[^ ^180^ ^]^ In addition to the amino acids, methanol, and ethanol were also produced. However, further research in this field is missing in the literature.

### 3.3 Photoelectric Conversion of CH_4_

Combining photocatalysis and electrocatalysis resulting in photoelectrode (photoelectric or photoelectrochemical (PEC) technique) is an interesting technique that can provide additional energy to meet the energy demand for breaking the inert C—H bonds in CH_4_. In this technique, methane oxidation occurs at the interface of the photoanode and electrolyte by the action of both irradiated light and applied potential. Photoelectrocatalyst can play important role to oxidize methane to oxygenates selectively at low temperatures as it has the advantage of monitoring the applied potential. We are not going to discuss it in details as it was coved in other review at length.^[^ ^93^ ^]^

### 3.4 Photochemical Processes for CH_4_ Activation

Activation of the C—H bonds in CH_4_ only by light, especially deep UV radiation is another approach for CH_4_ oxidation. This section is devoted exclusively to photochemical conversion, i.e., conversion carried out by photoroutes and chemicalroutes. Photochemical conversion is very convenient and economical because it requires only light. Although thus far the least explored of the categories, photochemical processes could be a promising alternative for the conversion of relatively inert CH_4_ to oxygenates under mild conditions without using a catalyst. Initial attempts were made in Japan at Yamaguchi University, where Ogura and Kataoka developed oxidative conversion of CH_4_ to alcohols, acids and ketones by a photochemical reaction with water vapor at atmospheric pressure and temperatures below 100 °C.^[^ ^76^ ^]^ To carry out such oxidation, CH_4_ and water vapor were fed into a reaction chamber. The gas mixture was exposed to a 20 W low pressure mercury lamp with illumination wavelengths of 185 and 254 nm. The water underwent dissociation by UV light according to the following reactions

(11) $$\begin{array}{c} H_{2}O\to H+•\mathrm{OH} \end{array}$$

(12) $$\begin{array}{c} H_{2}O\to H_{2}+O \end{array}$$

Successively, each hydroxyl radical abstracted a hydrogen atom from CH_4_ to initiate its conversion and generated a methyl radical, which then reacted with a water molecule to give methanol as shown below

(13) $$\begin{array}{c} CH_{4}+•\mathrm{OH}\to •CH_{3}+H_{2}O \end{array}$$

(14) $$\begin{array}{c} •CH_{3}+H_{2}O\to CH_{3}\mathrm{OH}+H \end{array}$$

**Scheme 2:**
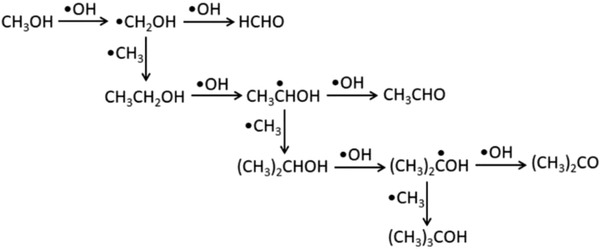
A generalized scheme to obtain different products according to Ogura and Kataoka. Reproduced with permission.^[^ ^76^ ^]^ Copyright 1988, Elsevier.

Methanol was the major product, with a selectivity of ≈70% at 90 °C. Higher species, such as formic acid, ethanol, formaldehyde, acetone, and acetic acid were formed with selectivity of 11%, 5%, 5%, 4%, and 3%, respectively, which were possibly originated from methanol. Notably, no higher alkanes were formed and the coupling of methyl radicals was insignificant. Large amounts of acetic acid and formic acid were formed with the addition of oxygen gas to the reaction mixture. A generalized reaction pathway for the generation of different products is depicted in **Scheme** **2**. A similar work described oxidative CH_4_ conversion by photolysis in the presence of water vapor and air at 100 °C and atmospheric pressure.^[^ ^181^ ^]^ Methane conversion occurred in the range of 4–16%, with selectivity for methanol was over 33%.

### 3.5 Photocatalytic Oxidation of CH_4_ with H_2_O_2_

**Figure 15:**
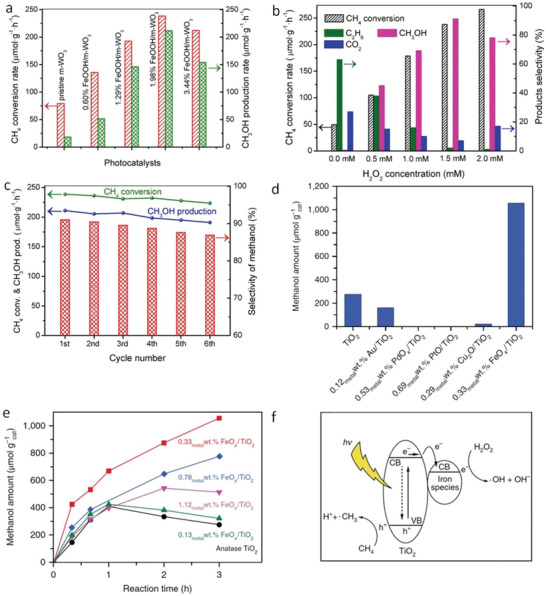
a) CH_4_ conversion rate and methanol production rate over different catalysts, b) methane conversion rate, and product selectivity over 1.98% FeOOH/m‐WO_3_ as the concentration of H_2_O_2_ varied. c) Methane conversion, methanol production, and selectivity over 1.98% FeOOH/m‐WO_3_ in different cycles. Reproduced with permission.^[^ ^155^ ^]^ Copyright 2020, Elsevier. d) Methanol production over different catalysts and e) over different amount of FeO*_x_*‐loaded TiO_2_. f) Photocatalytic mechanism. Reproduced with permission.^[^ ^158^ ^]^ Copyright 2018, the authors, published by Springer Nature Limited.

Hydrogen peroxide is a strong and green oxidizing agent that was usually used for partial oxidation of CH_4_ in thermocatalysis. However, it can be used for photocatalytic oxidation of CH_4._ Methane activation usually proceeds with the formation of •CH_3_ radicals that often undergo various reactions. This is the main reason for poor product selectivity in methane oxidation. Nonetheless, Yang et al. engineered mesoporous WO_3_ (m‐WO_3_) with amorphous FeOOH (FeOOH/m‐WO_3_) and reported photocatalytic partial oxidation of methane in the presence of H_2_O_2_.^[^ ^155^ ^]^ The CH_4_ conversion rate and product selectivity depended on the composition of the catalyst and the amount of H_2_O_2_ used. The catalyst 1.98% FeOOH/m‐WO_3_ exhibited the highest methane conversion rate (4.68% and 238.6 µmol g^−1^ h^−1^) as well as methanol production rate (211.2 µmol g^−1^ h^−1^) (**Figure** **15**a). Figure 15b shows that with the increasing concentration of H_2_O_2_ methane conversion rate increased. However, methanol production rate reached a maximum when the concentration of H_2_O_2_ was 1.5 mmol. More concentration of H_2_O_2_ led to decreased MeOH production rate and increased CO_2_ amount. The optimized catalyst achieved 91.0% selectivity for methanol production. The catalyst displayed considerable stability for up to six cycles (Figure 15c). Methane conversion rate decreased slightly from 238.6 to 223.7 µmol g^−1^ h^−1^ in the sixth cycle. MeOH production rate decreased from 211.2 µmol g^−1^ h^−1^ in the first cycle to 191.8 µmol g^−1^ h^−1^ in the sixth cycle. The observed decrease in methane conversion rate and methanol production rate was due to the slight decrease in Fe content after sixth cycle (1.19 wt% against 1.25 wt% for the fresh catalyst) as evidenced by inductively coupled plasma atomic emission spectroscopic (ICP–AES) data. Moreover, XPS did not show any obvious change in the chemical state of the fresh and the used catalyst. In a proposed mechanism, methane was activated to •CH_3_ radicals by the photo‐generated holes at the valence band of m‐WO_3_, while •OH radicals were generated by the decomposition of H_2_O_2_ by the photoelectrons of FeOOH. These radicals then combined to produce methanol selectively. On the other hand, Xie et al. reported FeO*_x_* anchored TiO_2_ photocatalyst for selective oxidation of CH_4_ to MeOH in the presence of H_2_O_2_ at room temperature and atmospheric pressure.^[^ ^158^ ^]^ The authors studied several metal oxides and noble metals and established that it was the iron oxide that was the most active for this transformation. A CH_4_ conversion rate of 15% was reported. Results on different metal oxide loaded on TiO_2_ showed that a maximum methanol production was achieved on FeO*_x_* loaded TiO_2_ (Figure 15d,e). A maximum yield for methanol production of 1056 µmol g^−1^ of catalyst was obtained on 0.33 wt% FeO*_x_*/TiO_2_. Notably, in this work the authors achieved high selectivity for alcohol (> 97%), wherein the selectivity for methanol generation was >90% with the optimized catalyst. The optimized photocatalyst showed good stability for three runs without any loss of catalytic activity. Proposed mechanism for photocatalytic methane oxidation to methanol is shown in Figure 15f.

### 3.6 Other Processes for CH_4_ Oxidation

Electric fields can be used to influence catalytic activity and selectivity of chemical reactions involving polarizable system, such as MeOH and metal/metal oxide support.^[^ ^182^ ^]^ External electric field can influence the adsorption energies of adsorbates on catalyst surface by changing the internal electric field of the catalyst (support) and adsorbates. Recently, through DFT calculations, Yeh et al. demonstrated that a positive electric field enhanced the oxidation of CH_4_ to HCHO by O_2_ over the oxygen‐rich (110) surface of IrO_2_.^[^ ^183^ ^]^ External electric field can suppress methane reforming temperature considerably.^[^ ^184^ ^]^ In addition, plasma can oxidize methane as can be seen in the following review.^[^ ^185^ ^]^ Nevertheless, Wang et al. described plasma assisted synthesis of oxygenates from CH_4_ and CO_2_ in one step at 30 °C and atmospheric pressure.^[^ ^186^ ^]^ The total selectivity to oxygenates (CH_3_COOH, MeOH, EtOH, and HCHO) was 59.1% in which the major product was CH_3_COOH for which the selectivity was 33.7%. Notably the combination of plasma with the catalyst Cu/*γ*‐Al_2_O_3_ increased acetic acid selectivity to 40.2%.

## 4 Conclusions and Future Perspectives

In order to build a better future and to cope with the environmental issues, we need to look for alternative energy sources other than the traditional fossil fuel, which releases considerable amount of CO_2_ and causes global warming. In this endeavor, we can turn our attention to CH_4_, the main constituent of natural gas that supplies ≈21% of world's energy demand. On combustion, CH_4_ releases a large amount of energy with minimum emission of CO_2_. Activation of CH_4_ is of remarkable importance, because this process will warrant future supplies of energy and fuel. It is also highly desirable for the synthesis of fine chemicals and pharmaceutical products. Unfortunately, the activation process faces kinetic limitations that lead to the formation of CO_2_ or poor product yields. Significant research effort has been made thus far for CH_4_ oxidation. However, efficient and well‐controlled CH_4_ oxidation process under mild conditions remains challenging. Many results are at an early stage and far from industrial applications due to the low conversion efficiency, slow rate of reactions and lack of economic competitiveness. Improvement of these techniques is expected to significantly ameliorate global environmental issues, as well as offer alternative energy sources. Fortunately, all these studies have led to progress in the fundamental knowledge of CH_4_ oxidation, catalyst design, syntheses, in situ and ex situ characterization and theoretical studies. Nevertheless, it is important to design more efficient catalysts that offer efficient conversion and desired selectivity for CH_4_ conversion. In the following paragraphs, we demonstrate a few research directions for CH_4_ oxidation that researchers might like to especially engage in to realize significant progress in future.

Electrochemical techniques are fascinating for the CH_4_ oxidation at low temperatures to produce value‐added chemicals. However, here main issue is the low solubility of CH_4_ in aqueous solution at standard conditions. This challenge can be overcome by adopting the following approaches. i) We can employ GDEs in the electrolyte. CH_4_ gas can be introduced into the active site of the electrolyte through a gas diffusion layer in the GDEs. The continuous supply of CH_4_ into the GDEs is expected to increase CH_4_ oxidation current that may fulfill the requirements for industrial applications, over the conventional submerged electrodes. ii) The solubility of CH_4_ could also be increased by increasing the pressure and decreasing the temperature of the electrolyte. iii) Using appropriate organic solvents could be an interesting alternative to circumvent the low solubility problem of CH_4_ in water. Organic solvents may lead to the formation of different products than those obtained in aqueous electrolytes. Additionally, organic solvents are less susceptible to undergo electrocatalytic oxidation unlike water. Thus, the issue of water oxidation (oxygen evolution that may damage the electrocatalyst by oxidation) could be avoided by using aprotic organic solvents. In fact, this area of research was not paid much attention so far and is awaiting further exploration.

Looking for electrocatalytic CH_4_ oxidation processes that lead C2/2+ products is another important direction, which must be considered by researcher, because it will add more value to the oxidation. Low electrical conductivity of the electrocatalysts is another challenge that increases charge transfer resistance at the interface. It can be greatly improved by designing composites with carbon‐based materials. Doping foreign elements into the active electrocatalysts not only alters the electronic properties of the latter but also enhances their electrical conductivity that improves catalytic activity. Another important aspect of electrocatalytic CH_4_ oxidation is the identification of the rate determining step (RDS) for the development of electrocatalysts. Charge transfer mechanism investigation will help to identify the RDS and overall, the development of electrocatalyst. In this regard, in situ and in operando spectroscopy and other in situ characterizations (such as, TEM, X‐ray diffraction, infra‐red spectroscopy) along with DFT calculations should be intensively performed to understand and engineer the existing electrocatalysts and discover new ones.

Photocatalysis is another promising technology for CH_4_ oxidation at low temperatures. The most important part of such research is to develop an efficient photocatalyst that can absorb light (preferably visible) efficiently and generate charge carriers owning sufficient energy to drive CH_4_ oxidation. This issue can be solved by designing a composite catalyst system, as a single photocatalyst often cannot satisfy all these conditions. A possible way out is to design a Z‐scheme photocatalyst system that has stronger ability to drive a photocatalytic reaction, unlike a conventional type II heterojunction. In many photocatalytic CH_4_ oxidation processes, hydroxyl radicals are the main intermediates. Unfortunately, due to the high reactivity of hydroxyl radicals, selectivity is often compromised. Controlled release of hydroxyl radicals could be a possible solution. Another important research direction is to employ PEC devices for CH_4_ oxidation to valuable organic compounds as demonstrated by Jin in a recent editorial.^[^ ^187^ ^]^ Recently, Grätzel and co‐workers. used PEC cell to synthesize high‐value added organics from arene C—H amination.^[^ ^188^ ^]^ In addition, H_2_O_2_ production by PEC recently attracted much interest.^[^ ^189^ ^]^ Such in situ generated H_2_O_2_ could be used for CH_4_ oxidation. These proposition and experimental evidence indicate prospects of PEC devices for CH_4_ activation where researchers are strongly recommended to pay attention. Considering these factors may lead to a photocatalyst or PEC system design that can oxidize CH_4_ proficiently for durable and practical applications in industry. Moreover, looking for highly active, stable and more selective catalysts is urgently necessary to make the CH_4_ activation process more economical.

Another research direction we would like to emphasize particularly is to use metal–organic framework (MOF) for both electrocatalytic and photocatalytic oxidation of CH_4_. The intrinsic characteristics of MOF would enable adsorption of large amount of CH_4_ onto the catalyst surface. Moreover, the organic moiety of MOF may be favorable for CH_4_ adsorption. Therefore, it would be interesting to see the catalytic properties of MOF for CH_4_ oxidation. Developing composite catalysts with MOFs and other catalysts may be a good proposition to improve catalytic efficiency. Nanocomposites of metal single atoms on porous carbon‐based supports can be used for photocatalyst and electrocatalyst for CH_4_ oxidation. The ultimate goal is to design high efficient, highly selective, durable, and cost‐effective photocatalysts and electrocatalysts for CH_4_ oxidation.

Long‐term stability and durability of the catalyst and selectivity of product are two critical issues for methane oxidation in both electrocatalysis and photocatalysis. Engineering the interface of a heterostructure can improve long‐term stability and durability of a catalyst by minimizing the agglomeration of the particles. Such engineering also may be able to minimize the coking on the catalyst surface. In this regard, very low concentration or single atoms of the active catalyst over the support is interesting. On the other hand, thorough understanding of the mechanism of a catalytic process by theoretical and experimental studies will enable us to improve selectivity of a desired product. Controlling the pressure of CH_4_ on the catalyst surface can improve selectivity. In this regard, a GDE can be used. Proper electrochemical cell design, electrolyte, reaction conditions, such as temperature, pressure, etc. and the choice of oxidizing agents also play important role in product selectivity.

Finally, technical and economic analyses that include cost of device manufacturing, carbon and energy efficiencies and product purification and distribution costs for methane oxidation via electrocatalytic and photocatalytic process needs to be considered. Computer‐based simulations are necessary to assess the performance of each process for a desired product. Very recently, Alsuhaibani et al. demonstrated that the reduction in reaction pressure of the reactor that produced methanol from shale gas resulted in substantial improvement of the profit of a plant with capacity of 2.1 × 10^6^ tons per year.^[^ ^190^ ^]^ Therefore, we can conclude that, from techno‐economic view point, methane oxidation to a desired product by electrocatalysis and photocatalysis in industrial scale is expected to be economically viable.

## Conflict of Interest

The authors declare no conflict of interest.
